# Pre-aggregation kinetics and intermediates of α-synuclein monitored by the ESIPT probe 7MFE

**DOI:** 10.1007/s00249-017-1272-0

**Published:** 2017-12-18

**Authors:** Jonathan A. Fauerbach, Thomas M. Jovin

**Affiliations:** 1 Miltenyi Biotec GmbH, Friedrich-Ebert Str. 42, 51429 Bergisch-Gladbach, Germany; 2 Laboratory of Cellular Dynamics, Max Planck Institute for Biophysical Chemistry, Am Fassberg 11, 37077 Göttingen, Germany

**Keywords:** Amyloid, Aggregation kinetics, Oligomers, Supramolecular intermediates, MFC, 3-Hydroxychromones

## Abstract

The defining feature of the extensive family of amyloid diseases is the formation of networks of entangled elongated protein fibrils and amorphous aggregates exhibiting crossed β-sheet secondary structure. The time course of amyloid conversion has been studied extensively in vitro with the proteins involved in the neurodegenerative pathology of Parkinson’s disease (α-synuclein), Alzheimer’s disease (Tau) and Huntington’s disease (Huntingtin). Although much is known about the thermodynamics and kinetics of the transition from a soluble, intrinsically disordered monomer to the fibrillar end state, the putative oligomeric intermediates, currently considered to be the major initiators of cellular toxicity, are as yet poorly defined. We have detected and characterized amyloid precursors by monitoring AS aggregation with ESIPT (excited state intramolecular protein transfer) probes, one of which, 7MFE [7-(3-maleimido-*N*-propanamide)-2-(4-diethyaminophenyl)-3-hydroxychromone], is introduced here and compared with a related compound, 6MFC, used previously. A series of 140 spectra for sparsely labeled AS was acquired during the course of aggregation, and resolved into the relative contributions (spectra, intensities) of discrete molecular species including the monomeric, fibrillar, and ensemble of intermediate forms. Based on these findings, a kinetic scheme was devised to simulate progress curves as a function of key parameters. An essential feature of the model, one not previously invoked in schemes of amyloid aggregation, is the catalysis of molecular *fuzziness* by discrete colloidal nanoparticles arising spontaneously via monomer condensation upon exposure of AS to ≥ 37 °C.

**Electronic supplementary material:**

The online version of this article (10.1007/s00249-017-1272-0) contains supplementary material, which is available to authorized users.

## Introduction

Alpha-synuclein (α-synuclein, AS) is a small (140 aa, 14.5 kDa), primarily presynaptic neuronal protein, noted for its presumed (Lashuel et al. 2013; Rocha et al. 2017) neuropathological role in numerous neurodegenerative conditions, denoted synucleopathies. The notable example is Parkinson’s disease (PD) (Williams-Gray and Worth 2016). PD is primarily (yet not exclusively) a movement disorder, featuring a loss of functional, viable dopamine mediating neurons in the brain stem, in which focal intracellular accumulations (Lewy bodies, Lewy neurites) of aggregated AS and other proteins are characteristic. The mechanisms of AS-dependent toxicity and their therapeutic implications are controversial (Burbulla et al. 2017; Burre et al. 2017; Wong and Krainc 2017). Designated as a protein-chameleon both from a structural and functional point-of-view, the highly pliable structure of AS is extremely sensitive to environmental conditions (Uversky 2017). Upon incubation in vitro, intrinsically disordered (ID), albeit not completely unfolded monomeric AS, undergoes intermolecular self-assembly in a process referred to in the field of protein-folding diseases (Valastyan and Lindquist 2014) as misfolding (Breydo et al. 2016) and aberrant aggregation.^1^ The end products are highly ordered and elongated fibrils characterized by regular in-register cross-β-sheets, with hydrophobic core residues and other distinctive features of amyloid secondary and tertiary structure (Tuttle et al. 2016). These mature amyloid fibrils (in our nomenclature *mature amyloid fibrils*, *mafs* (Fauerbach et al. 2012)) are a common defining feature exhibited by numerous proteins in the extensive family of protein-folding amyloid diseases.

Great attention has been given to the nucleation and propagation mechanisms involved in protein fibrillation (Cabriolu et al. 2010; Michaels et al. 2017). In particular, the elucidation of how monomeric AS arranges into *mafs* has been considered of high priority inasmuch as it constitutes the molecular basis by which key reaction steps and species (cytotoxic supramolecular, fibrillar, and non-fibrillar AS aggregates) can/could become unequivocally identified and potentially isolated. This would provide a molecular therapeutic strategy, for example based on chemical, antibody based aggregation inhibitors, or modulators of conformation (Gonzalez-Lizarraga et al. 2017), to delay, halt or even revert the loss-of-function and gain-of-toxic structure and function exhibited by AS in amyloidogenic states.

The extensive theoretical treatments of nucleated fibrillation facilitate data analysis and interpretation, elaborated on and reviewed in Bentea et al. (2017), Kumar et al. (2017a) and Michaels et al. (2017). However, these schemes do not explicitly accommodate the genesis, time course and nature of intermediates—currently presumed to be the major initiators of cellular toxicity—in the transition from a soluble ID monomer to the highly ordered cross-β-sheet fibrillar end state. However, an appropriate formalism is required for the elucidation of structure/function relationships. For example, a recent study (Iljina et al. 2016) indicated that despite the presumption that pathological prion-like spreading of toxic AS aggregates in PD operates via templated seeding, the required concentrations are much higher than those sufficing for oligomer induced cellular stress. The implication is that transient oligomeric species present at even vanishing concentrations and/or for short periods of time may be essential to an understanding and therapeutic exploitation of the overall amyloid mechanism. Simple two-step conformational nucleation-polymerization transitions (the Finke–Watzky model) are in this respect insufficient. They may indeed serve to empirically define the sigmoidal time course of fibrillation assessed with external probes such as thioflavin T (ThioT), yet both the initial process of primary nucleation and the generation of intermediates are neglected.

The amyloid oligomer literature, even restricted to AS, is vast (Hong et al. 2011; Uversky 2017) and has been recently reviewed (Cremades et al. 2017). The latter authors cite three mechanisms for the transition from the monomer to oligomeric states of association: (1) nucleation–polymerization; (2) nucleation–conversion–polymerization involving an intermediate species with pre-amyloid beta-structure; and (3) coalescence of monomer and maturation to an oligomer with β-sheet structure capable of end-elongation by templating or induced fit. The authors further provide an extensive classification of oligomers according to the mode of formation: isolation during in vitro fibrillation; stabilization by lyophilization; stabilization by chemical compounds, including metals; stabilization by chemical modification; generation by fibrillar disaggregation; and identification from in vivo material. The main limitation of studies based on such materials is evident. Steady-state thermodynamic equilibria, as well as kinetic steps dictate the time course of any intermediate. Fibrillation is largely irreversible, such that the monomer concentration after the onset of fibril formation will necessarily diminish to levels below the *K*_d_s of non-covalent protein association. As a consequence, almost any conceivable form of isolation will lead to dissociation of intermediates that form only at the high (typically > 100 µM) monomer concentrations generally selected for in vitro studies. A further complication arises from the myriad parameters that influence the reaction kinetic and yields, including the relation between nucleation and growth (Buell et al. 2014), solution composition, ionic strength, and pH; cosolvents; temperature; mechanical agitation (Batzli and Love 2015); intentional and/or inadvertent protein modification; molecular crowding agents; and the presence of organelles and/or membranes. These dependencies not only determine the great degree of observed fibrillar and oligomeric polymorphism (Cremades et al. 2017; Fauerbach et al. 2012; Uversky 2017) but are manifested already in the monomer, the solution structure of which depends greatly on pH and ionic strength (Bai et al. 2016), as well as on the perturbations of long range interactions, for example by introduction of the disease-related site-specific mutations (Ranjan and Kumar 2017). Furthermore, it appears that many features common to amyloid proteins, especially of the ID class (Uversky 2017), are more generally applicable to almost any protein under given physiological or denaturing conditions, leading to the postulate that *all* proteins can enter the *amyloid state* (Eisenberg and Jucker 2012; Knowles et al. 2014). Nonetheless, one can anticipate that nucleation and oligomerization are more system (protein) specific.

Numerous spectroscopic (fluorescence, NMR, EPR, IR, X-ray scattering, light scattering), microscopy (fluorescence: steady-state and time-resolved, FCS, FRET, single molecule, microfluidics; SHG; Raman; AFM and STM; cryo-EM), chromatography and other biophysical methods have been applied to the study of amyloid protein aggregation. Fluorescence based methods are the most accessible and in any ways versatile, due to their inherent high sensitivity, and the availability of a wide array of intrinsic, extrinsic, and expression probes with environmental sensitivity (Bertoncini and Celej 2011; Eliezer 2016; Haney et al. 2016; Lindgren and Hammarstrom 2010; Pinotsi et al. 2014; Plotegher et al. 2014; van Ham et al. 2010). An important issue is the effect of direct protein labeling, particularly probe/protein stoichiometry, on the aggregation process and product morphology (Mucibabic et al. 2016; Thirunavukkuarasu et al. 2008; Yushchenko et al. 2010). Our own studies of the early (as well as later) stages of AS aggregation in vitro have been based on the use of various members of the *N*-arylaminonaphthalene sulfonate family (Celej et al. 2008), pyrene (Thirunavukkuarasu et al. 2008), and ESIPT probes (Celej et al. 2009; Yushchenko et al. 2010), exploiting the readily available fluorescence parameters (spectra, intensity, emission anisotropy, FRET, lifetime, high-resolution microscopy) and comparing them with the standard ThioT readout. The latter is unfortunately insensitive to the formation of pre-amyloid molecular species due to its high background and binding selectivity for *mafs* (Kumar et al. 2017b).

**Fig. 1:**
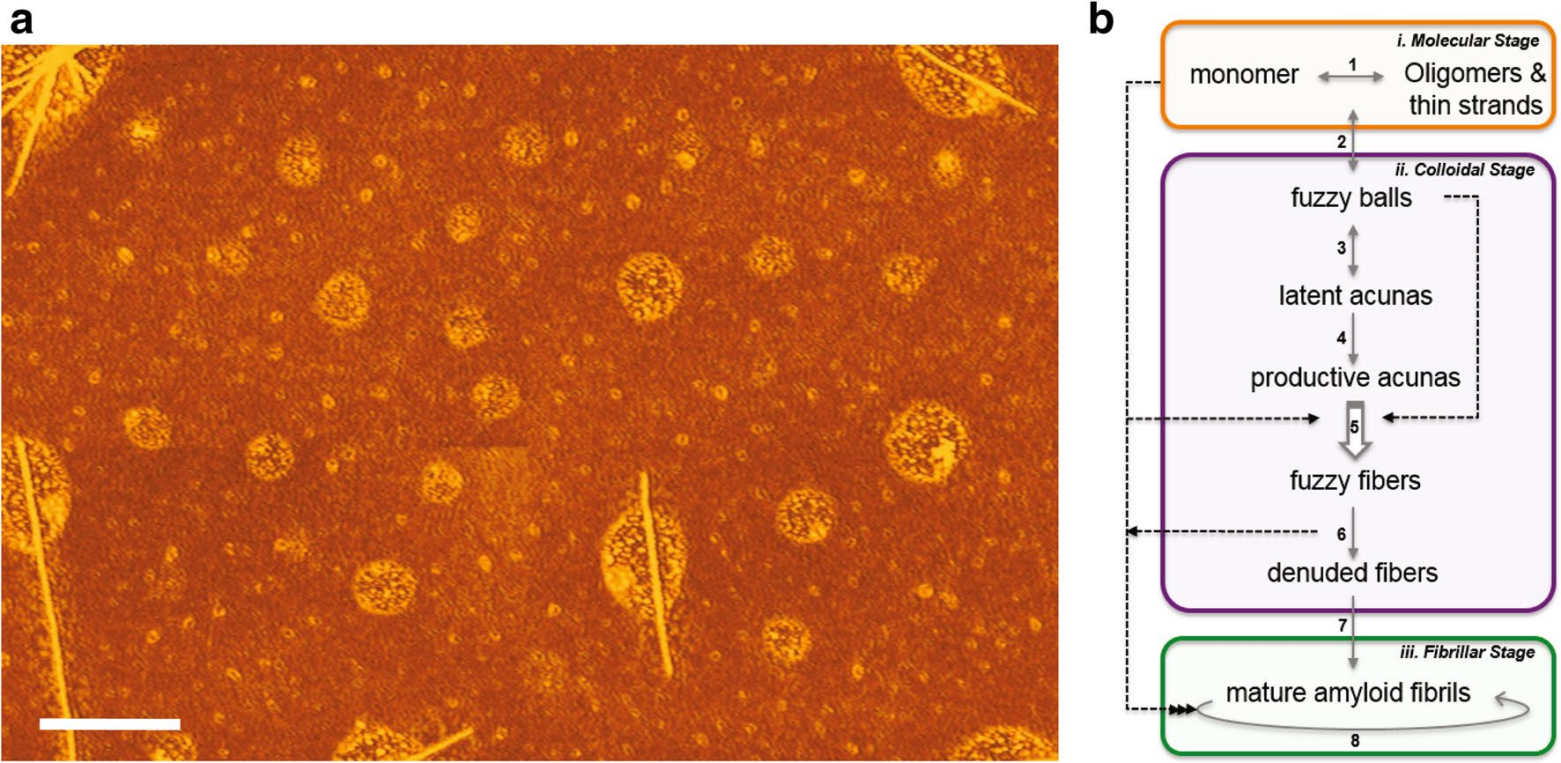
**a** AFM image of a field containing a variety of co-existing AS colloidal oligomers, intermediates, supramolecular structures and *fuzzy* deposited over a layer of monomeric and oligomeric AS. **b** Sequential aggregation scheme (SAS) for α-synuclein, summarized and adapted from (Fauerbach et al. 2012). The scheme depicts molecular, colloidal, and fibrillar stages for AS species in terms of the distinct reaction steps (numbered). (1) oligomerization (monomeric AS in equilibrium with e.g. dimers, tetramers). (2) Colloidal condensation (*fuzzy and ball*). (3) Supramolecular *acunas*. (4) Fibril elongation via activated *productive acunas*. (5) Generation and release of *fuzzy fibers* (the open arrow indicates a template function of the productive *acunas*). (6) Loss of *fuzziness* (denuded fibers) and transformation to other prefibrillar structures. (7) Conformational transition to the amyloid fibrillar form. (8) Fibrillar growth by mechanical fragmentation and terminal extension via scavenging of monomers and oligomers

In 2010, we introduced the ESIPT probe MFC (herein 6MFC) as a protein polarity sensor reporter for continuous monitoring of AS folding and self-assembly during aggregation in vitro (Yushchenko et al. 2010). MFC, a member of 3-hydroxychromone fluorophore family pioneered by Klymchenko and Demchenko (2008), exhibits a dual-band emission spectrum, in which the position and intensity of the N* and T* bands provides a polarity-based fluorescent roadmap of AS aggregation. The A140C mutant of AS was labeled with MFC-maleimide, positioning the probe at the C-terminus. The 2% (labeled/wild type AS) protein was exposed to 37 °C under agitation and complete spectra were acquired at 30-min intervals. An increase in emission intensity was perceived as of 3–4 h of incubation, reaching a maximum in intensity by 16 h, at which time the classical ThioT assay performed in parallel corresponded to only ca. 20% of its maximum signal (achieved at 70 h); the ThioT signal was mirrored by a light scattering signal obtained at the excitation red edge. We concluded that the ESIPT probe was monitoring processes occurring during the conventional *lag time* inaccessible by ThioT. In these experiments, monomeric AS obtained after ultracentrifugation exhibited a dual-band spectra and a T*/N* ratio < 1, a value in good correspondence with the fluorescence signature of MFC in polar protic solvents, although the quantum yield (QY) was higher for the covalently protein-bound probe than free in solution, an indication that the C-terminal of AS was fairly water-exposed yet part of a partially folded molecule. During the reaction, the AS self-assembled into supramolecular structures, culminating in the formation of *mafs* (Fig. 1). We interpreted the accompanying fluorescence manifestations (increase in the fluorescent signals of both the N* and T* bands and thus in QY, large increase in T*/N* to 8–10, bathochromic shift of the T* band) as indicative of a transfer of the probe to a less polar environment, corresponding closely to DMF according to a reference spectrum obtained for that solvent. Thus, despite the maintenance of the protein in bulk water, a transformation involving water extrusion and formation of intermediate structures was evident and further revealed by emission anisotropy and reduced polarity reported previously using ANS probes (Celej et al. 2008).

These studies were extended (Fauerbach et al. 2012) by visualizing the intermediate structures arising during AS aggregation with atomic force microscopy (AFM) and cryo-electron tomography (cryo-ET), exploiting the temporal guidance provided by the same 6MFC-AS probe. We identified a vast family of supramolecular structures (defined as *balls*, *acunas*, *fibrils* and *mafs*) and their sequential appearance during AS in vitro aggregation (Fig. 1). A particular significance was attached to a category of surface-bound AS oligomers, identified as *fuzzy* species, both by AFM and cryo-ET, and we postulated that they play a key role in recruiting monomeric AS from solution into transient supramolecular assemblies that mature into *mafs*. In particular, the *acuna* [*amiloide* + *cuna* (crib)] was identified as a supramolecular assembly constituting loci for the nucleation and maturation of fibrils. A pronounced temperature dependence of this process was established by determining that the progenitor structures were formed, but kinetically trapped at low temperature (4 °C). Extensive controls emphasized the transient nature of these supramolecular structures, which disintegrated upon reductions in ionic strength and concentration, and changes in pH [SI of (Fauerbach et al. 2012)]. A *Sequential Aggregation Scheme* (SAS) comprising molecular, colloidal, and fibrillar stages was derived from this study and is reproduced in Fig. 1b. *Acunas* have been observed in other publications but not identified as such (Gallea and Celej 2014).

In the present study, we report a more detailed view of amyloid precursor progression, particularly at its onset, obtained by monitoring AS aggregation with another, novel ESIPT probe, 7MFE [7-(3-maleimido-*N*-propanamide)-2-(4-diethyaminophenyl)-3-hydroxychromone (Fauerbach 2013). The FE molecule, the parent of 7MFE, was previously used by our group as an external triple-emission probe to distinguish structural features of *mafs* formed from wild-type AS, and disease-relevant familial mutants (A53T, A30P and E46K) (Celej et al. 2009). The H-bonded NH* species was observed and its contribution to the emission spectra quantified after proper spectral deconvolution. It was recognized that (a fraction of) the red-shifted NH* form presumably arises from the formation of intermolecular interactions with OH and NH side groups of the protein, confirming the intrinsic tendency of AS to form cross-H-bonds, the fundamental element of β-sheet structures. As in the case of 6MFC, the new 7MFE enables stable and unique labeling at given sites, thus providing more specific information about local microenvironment and structure. Spectra of AS covalently labeled with 7MFE or 6MFC were acquired during the course of aggregation, and resolved into the relative contributions (spectral, abundance) of apparent molecular species to the evolving population. A kinetic scheme was devised to simulate the progress curves as a function of relevant parameters. A key feature of the model, one not previously invoked in schemes of amyloid aggregation, is a catalytic role ascribed to the discrete colloidal nanoensembles (< 50 nm), arising by rapid monomer condensation at ≥ 37 °C, in the generation of the essential *fuzziness*. Autocatalysis is also a feature of fibril elongation.

In this communication, we present a quantitative treatment based on the data and concepts of Fig. 1 and the additional findings gained with the new 7MFE probe. Extension to the vast number and forms of aggregation mechanisms that can be found in the literature is beyond our intentions and scope. However, it is our expectation that the approach can be generalized.

## Methods and results

### ESIPT probes as polarity and H-bonding reporters of AS aggregation pathway

**Fig. 2:**
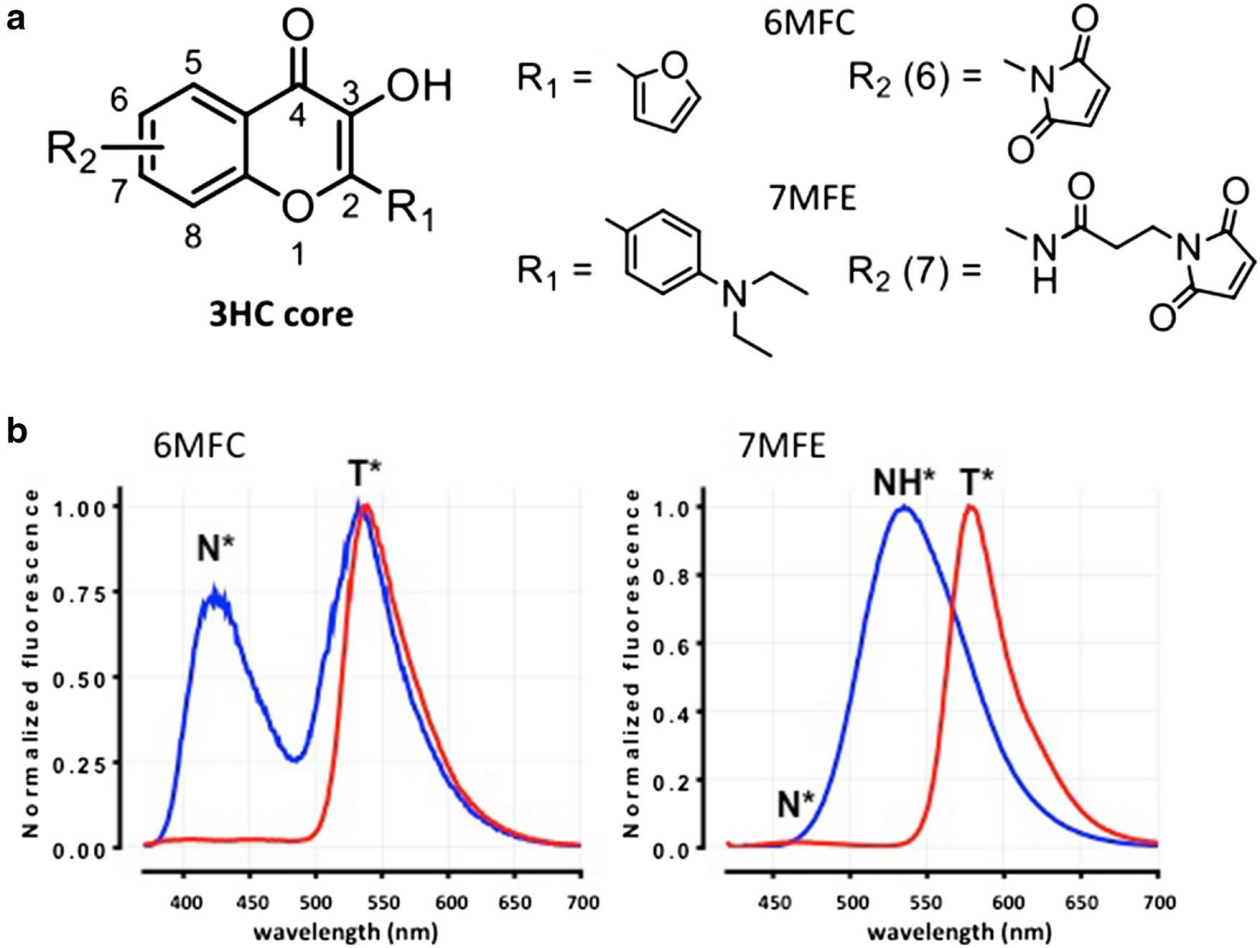
Chemical structure of the 3HC core (**a**) and spectra (**b**) of 6MFC and 7MFE. R_1_ and R_2_ indicate the corresponding substituents at position 2 and positions 6 and 7, respectively. Emission spectra in toluene (red) and methanol (blue) with excitation at 360 and 420 nm for 6MFC and 7MFE, respectively. The number, position, shape and relative intensities of bands depend on the polarity and H-bonding capacity of the probe microenvironment

**Fig. 3:**
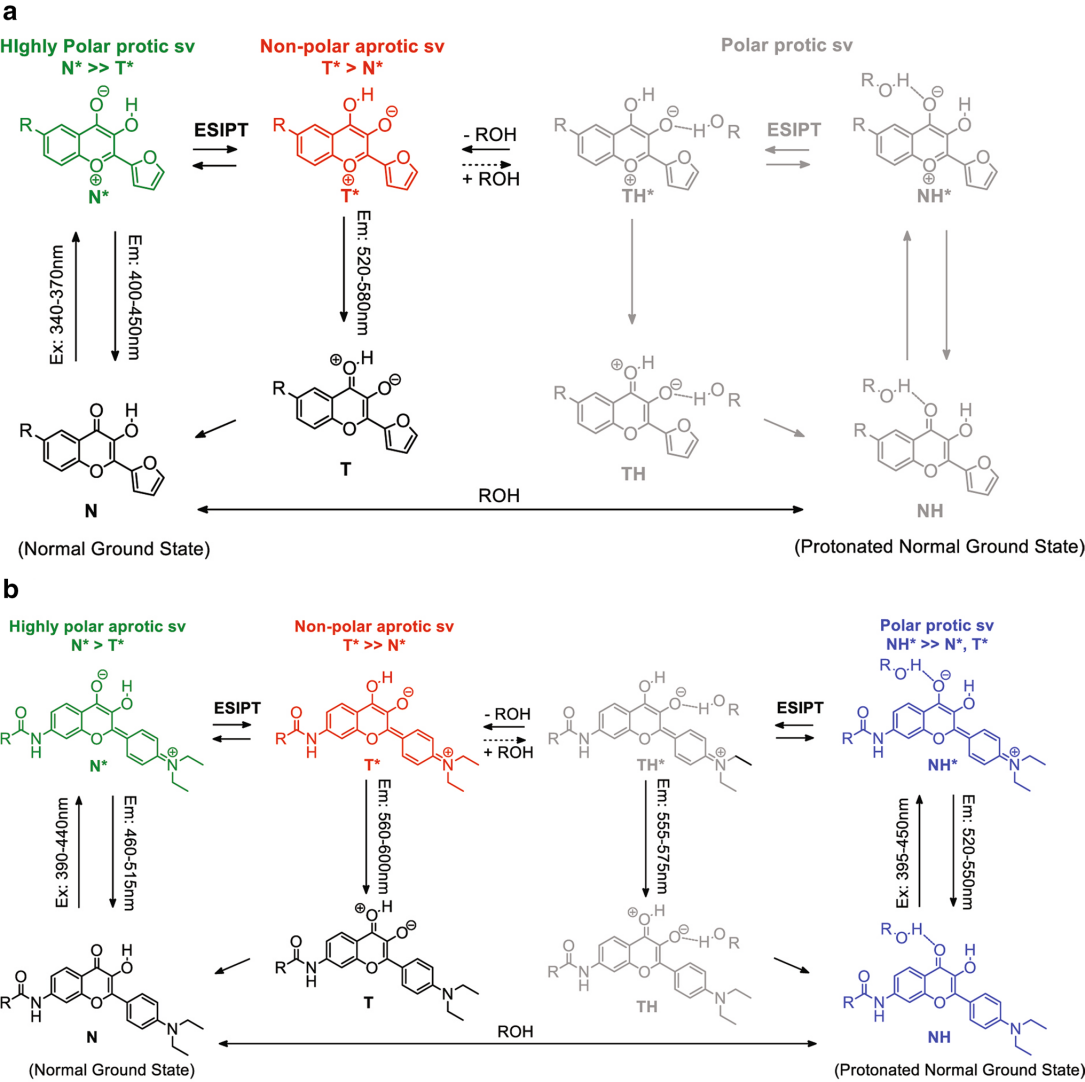
ESIPT scheme for **a** 6MFC and **b** 7MFE. The equilibria between N, T, NH and TH forms, as well as their excited states (asterisk) are represented. Excitation and emission wavelengths are indicated as ranges for each form. Both ESIPT and solvent intermolecular H-bonding equilibria are depicted. Colors serve as visual aids to help identify the predominant species with high emission intensity under different environments, chosen here due to their relevance to our study. The N* form (green) usually predominates with high intensity in polar protic solvents for 6MFC (**a**), and in polar aprotic solvents for 7MFE (**b**). The T* form (red) predominates with very high intensity in non-polar aprotic solvents for both 6MFC (**a**), and 7MFE (**b**). In polar and protic solvents, the NH* form predominates over the N* and T* forms for 7MFE (**b**), but is not significant for 6MFC (**a**) and is thus colored in gray. The TH* (gray) is either of very low intensity and/or poorly populated by ESIPT formation. It is undetectable for both probes (Kenfack et al. 2012, Klymchenko et al. 2003).

The synthesis and characterization of 7MFE (Fig. 2), the new reactive (thiol-reactive maleimide) probe of the 3-hydroxychromone (3HC) ESIPT family, are given in the ESM. 7MFE complements 6MFC in having a much greater sensitivity for H-bonding. Although both belong to the 3HC class of molecules, each has its own distinctive spectroscopic response to environmental changes in terms of polarity, hydration and H-bonding. In the 3HC structure, the OH group in position 3 forms an intramolecular H-bond with the oxygen of the adjacent carbonyl in position 4 (Fig. 3). Proton transfer results in two characteristic excited-state forms with distinct and well-separated emission bands, a normal (N*) and the ESIPT product tautomer (T*). The OH group also forms intermolecular bonds with proton H-bond acceptors and the carbonyl forms H-bonds with proton-donor groups, thereby reporting on the basicity and acidity, respectively, of the microenvironment (Giordano et al. 2012). The different spectral features derive from both the nature and position of substituents around the 3HC core (Klymchenko et al. 2003). These dictate the charge and electronic (re)arrangements upon excitation, reflected in degree of charge separation, electronic dipole orientation, energy levels of the excited species and acidity of the proton involved in ESIPT. The latter feature is the main determinant of the spectroscopic behavior. Although both probes have rich-electron donating groups located at position 2 (a furyl group in 6MFC and a *N*-diethylaminoaryl group in 7MFE, Fig. 2a), their hydrophobicity and ability for charge stabilization are quite different, rendering them attractive as a fairly orthogonal pair for monitoring polarity and hydration (H-bonding) at given locations in the protein sequence.

To rationalize the results obtained in studies of AS aggregation, we recorded the emission spectra of both ESIPT probes in different organic solvents, providing a calibration with predictive value; the data for toluene and methanol are shown in Fig. 2b. In non-polar aprotic solvents, such as toluene, both probes exhibit the dual-band ESIPT emission in which the short-wavelength and long-wavelength bands correspond to the emission of the normal (N*) and the tautomeric (T*) excited states, respectively. These bands are located at ~ 407 nm (hard to perceive) and 539 nm with a T*/N* ratio of ~ 40 for 6MFC, and at ~ 470 (also hard to perceive) and 580 nm with a T*/N* ratio of ~ 60 for 7MFE. A pronounced difference between the two probes is evident in their emission spectra in polar and particularly protic solvents, such as methanol (depicted in Fig. 2b) or water, the usual environment experienced by proteins and thus of greatest interest. The number of emission bands, and their positions, relative intensities and quantum yields vary dramatically upon transfer to methanol. 6MFC still exhibits a dual-band emission spectrum but with a bathochromic, hypochromic shift. The N* and T* bands are situated at ~ 423 and 535 nm and the T*/N* ratio is much lower (~ 1.3). In contrast, 7MFE in methanol still shows a single-band at ~ 535 nm. This feature, shared with a 4′-dialkylamino-3HF of similar structure, is particularly useful for sensing H-bonding, rendering the probes particularly attractive for studies of protein folding involving water extrusion and formation of regular H-bonded secondary structures, notably β-sheets. In these cases, the single band corresponds to an excited H-bonded normal ground state (NH*). The N and NH ground state exist in equilibrium and can be independently excited. The great utility of 7MFE-type molecules as probes of H-bonding is that they do not undergo proton transfer in the excited state and thus retain their individual emissions (Shynkar et al. 2004). In contrast, 6MFC was designed to avoid the formation and stabilization of H-bonded species thanks to its furyl substituent, establishing it as a superior polarity probe.

### Acquisition of emission spectral time series during aggregation

**Fig. 4:**
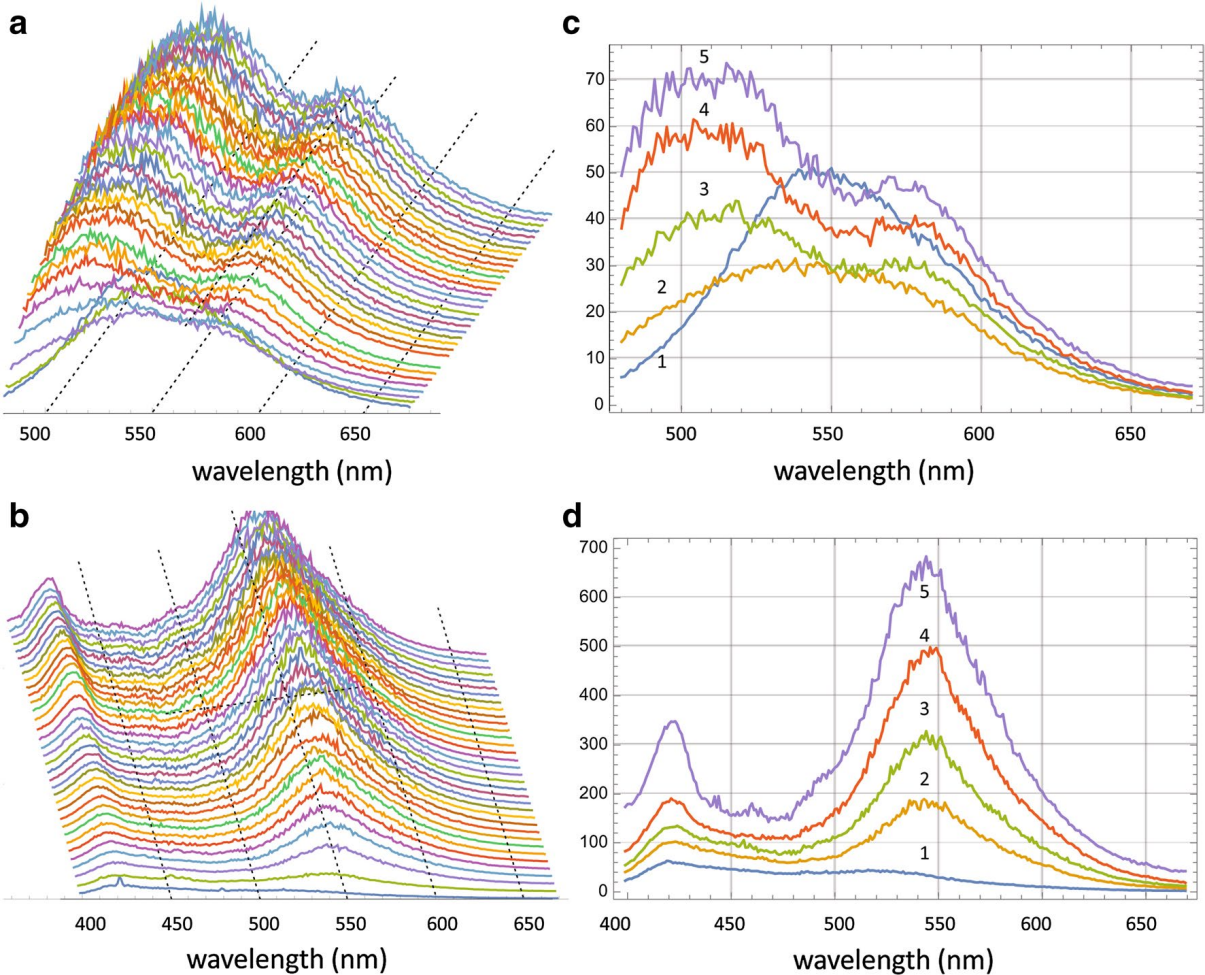
Emission spectra of 7MFE (**a**, **c**) and 6MFC (**b**, **d**) during the time course of AS aggregation. **a**, **b** Every fourth time point of the entire sequence is displayed. Selected time points 1–5 in **c**: 0, 4.5. 9.5, 29.5, 69.5 h. Selected time points 1–5 in **d**: 1, 4.5, 9.5, 19.5, 69.5 h

Using a similar experimental setup as that described previously (Yushchenko et al. 2010), two samples were prepared: (1) 98% wtAS + 2% AS-A18C-6MFC, and (2) 98% wtAS + 2% AS-A18C-7MFE, incubated at 37 °C under continuous agitation in a square cuvette, and excited at their respective maxima, 360 and 420 nm, respectively. Emission spectra were acquired at half hour intervals up to 70 h (140 time points). A 3D representation of the obtained experimental emission spectra is shown in Fig. 4.

**Fig. 5:**
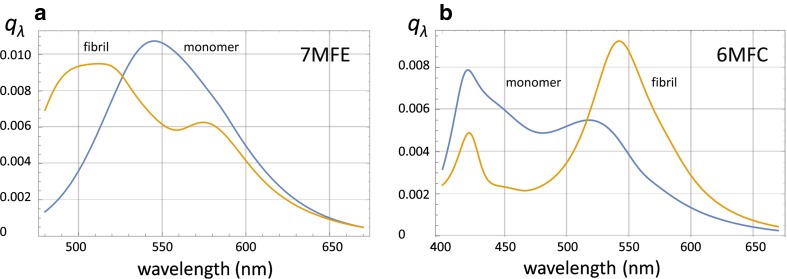
Spectral quantal emission distribution functions for the monomer (**sp1**) and fibril (**sp3,**) forms of A18C-AS labeled with **a** 7MFE or **b** 6MFC. Derived from the spectral data acquired during the aggregation reactions. The relative weights *w*3/*w*1 for 7MFE and 6MFC are 1.6 and 10.2, respectively

**Fig. 6:**
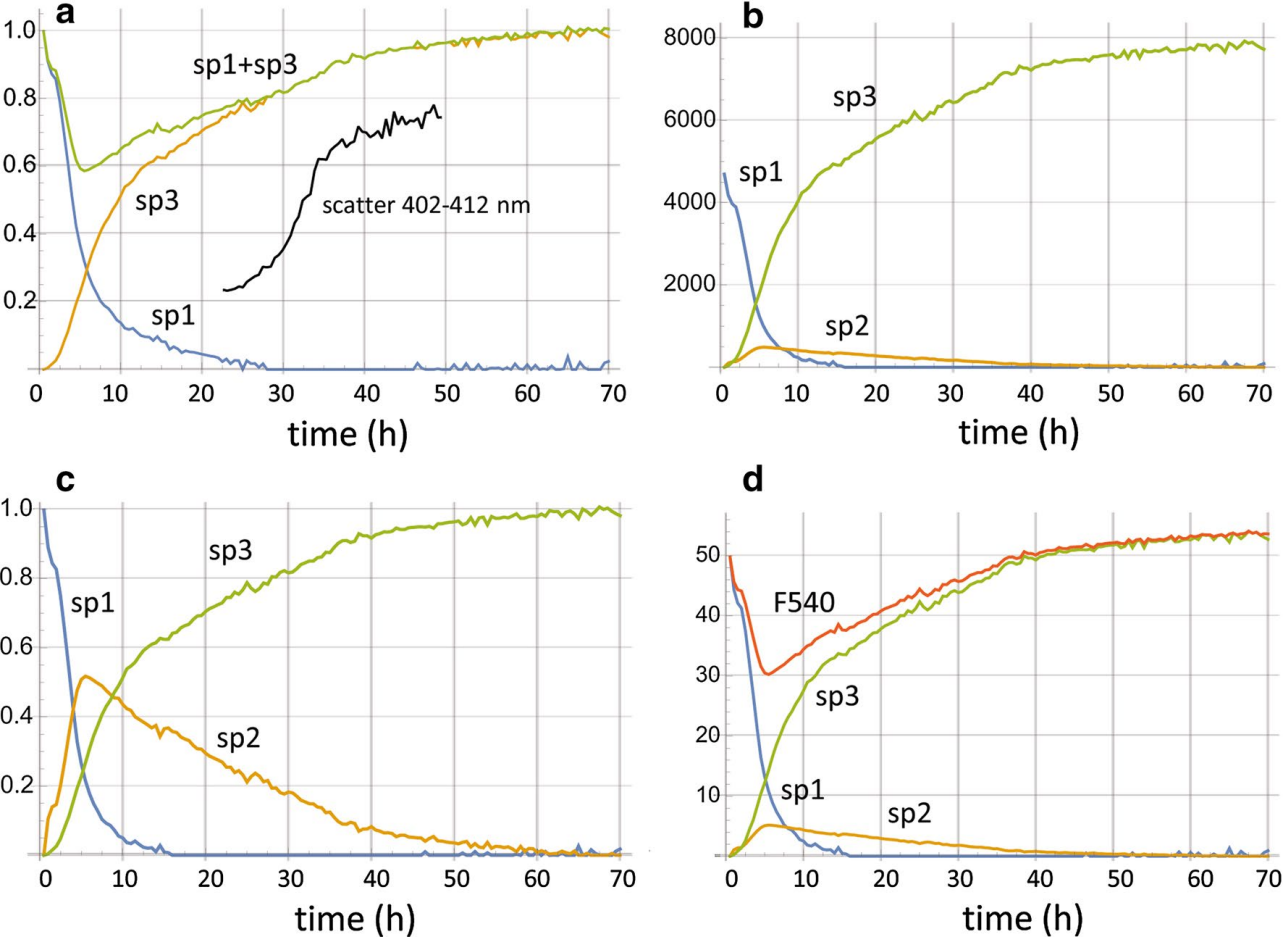
Time course of aggregation reaction monitored with 7MFE. **a** apparent contributions in fractional concentration units (*x*1, *x*3) to spectral signal from **sp1** (monomer spectrum, blue), and **sp3** (fibril spectrum, orange); green: sum; light scatter (inset) midpoint at ~ 33 h. **b** relative contributions to spectral signal by **sp1** (blue), **sp2** (intermediate with 0.2 relative quantum yield, orange), and **sp3** (green); **c** normalized contributions (in concentration units) to spectral signal by **sp1** (blue), **sp2** (orange), and **sp3** (green); the sum is = 1. **d** normalized contributions to fluorescence signal at 540 nm by **sp1** (blue), **sp2** (orange), and **sp3** (green); time course of signal at 540 nm (F540, red)

**Fig. 7:**
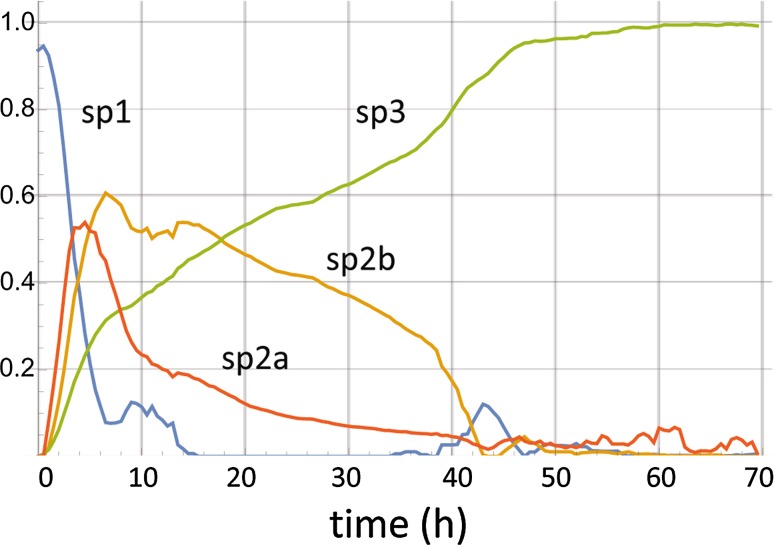
Time course of aggregation reaction monitored with 6MFC. In addition to **sp1** and **sp3**, two spectral components were assigned to the compartment of reaction intermediates. The light scattering signal (not shown) exhibited the same rapid increase at 40 h as **sp3**

**Fig. 8:**
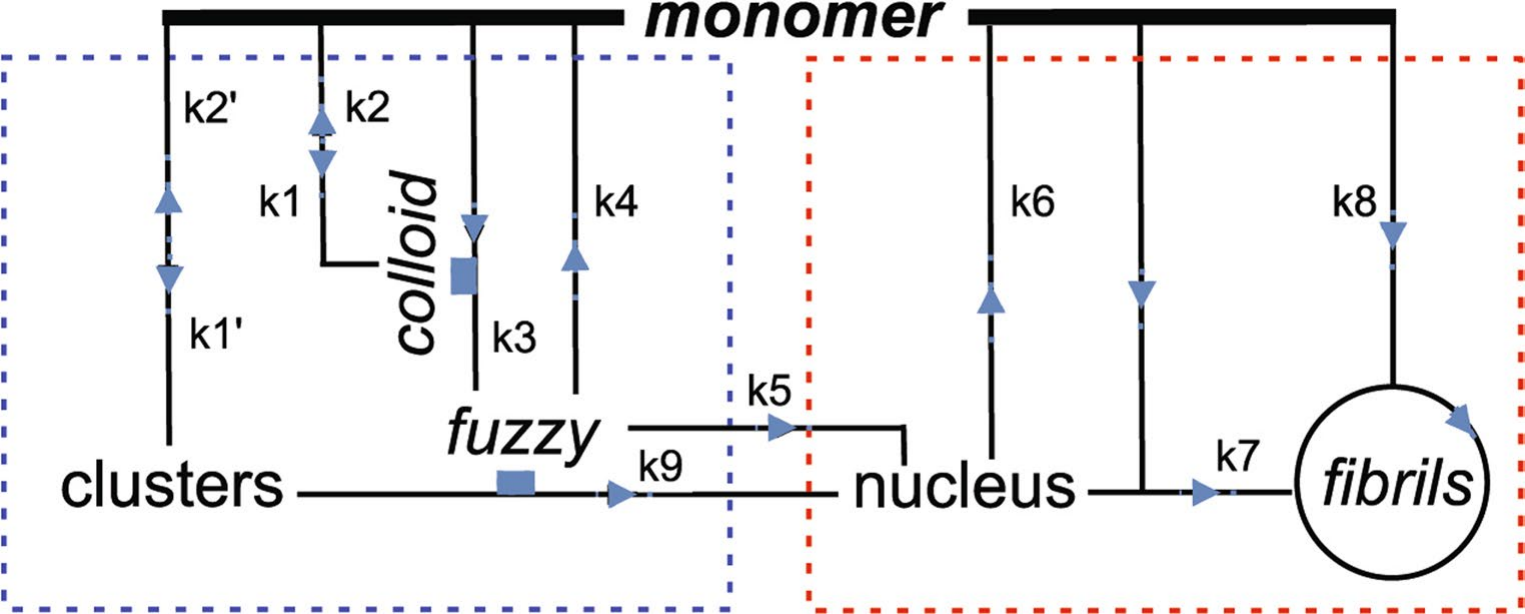
Concerted reaction scheme for AS progression from monomeric to fibrillar structures. Stippled blue domain: pre-amyloid manifold; stippled red domain: amyloid manifold. Clusters are associated oligomeric species. *Fuzzy* is an associated form of AS (see ESM) created on the surface of *colloid* [catalytic function denoted by italics and small blue rectangle) with a rate constant *k*3 (*colloid*)] and released, progressing via spontaneous conformational transition (*k*5) to an amyloid nucleus that is absorbed into the fibrillation cycle by end addition of monomer (*k*7). The formation of clusters, *colloid*, *fuzzy*, and nucleus are considered as concerted and assigned *n*1, *n*2, *p*, and *p* monomers units, respectively in the kinetic equations (ESM). A redistribution between supramolecular forms in the *fuzzy* compartment is not featured explicitly. *Fibrils* undergo autocatalytic expansion (*k*8, circle) involving secondary nucleation and fragmentation (not indicated). A potential parallel amyloid nucleation path involves *fuzzy*-catalyzed conversion of clusters to nucleus (*k*9) and conceivably (not shown) other supramolecular species. Clusters can also function as a monomer “reservoir, buffer”. Additional details are given in the ESM

In the AS aggregation reaction monitored by 6MFC, a dual-band emission spectrum was registered at time 0, with maxima located at 420 and 518 nm, and a T*/N* ratio of ~ 0.7 (Fig. 4d, spectrum 1 and Fig. 5b). The reaction proceeded with a continuous increase in total emission intensity, with the long-wavelength T* band progressively dominating over the N* band. Already at 4.5 h the T*/N* ratio was > 1 (Fig. 4d, spectrum 2), reaching a plateau between 60 and 70 h with a T*/N* value of ~ 1.9 (Fig. 4d, spectrum 5). For 7MFE the spectral evolution followed a completely different regime. At time 0, the probe exhibited a single-band spectrum centered at 544 nm (Fig. 4c, spectrum 1) and the shape was maintained during the initial 5 h. However, the intensity at certain wavelengths (e.g. 540 nm) decreased progressively (and linearly) from 0.5 to 5 h. Thereafter, the single band underwent a shape and intensity transformation, with a dual-band emission at ~ 528 and 572 nm of increasing intensity (Figs. 4a, c, 5, 6, 7, 8 h), and hypsochromic and bathochromic shifts, respectively, of the two bands (N*, 528 → 510 nm and T*, 572 → 575 nm (Fig. 4a, c, 10–37 h). Thereafter, the intensity achieved a plateau value that was maintained until 70 h. In contrast to 6MFC, the final T*/N* ratio was < 1 (Fig. 4a, c). The temporal spectral evolution and final T*/N* values of the two probes indicated a progressive decrease in polarity, which is treated further in the “Discussion”, and low or absent intermolecular H-bonding and/or shielding from the aqueous medium.

### Component analysis of the acquired spectra: Stage 1

In view of the pronounced spectral transitions during the reaction, we undertook a detailed analysis with the goal of extracting the spectral signatures of the important protein forms, including the elusive intermediates. The coupled ground and excited state equilibria of highly solvatochromic (Giordano et al. 2012) ESIPT dyes constitute a difficult challenge for spectral decomposition. We have previously applied the Siano-Metzler asymmetric log–normal function (alogn) to deconvolve combined excitation and emission spectra of an external ESIPT probe in defined solvents (Caarls et al. 2009). A set of conditions had to be fulfilled in order to achieve convergence. From the technical limitations of the spectrofluorimeter utilized in this study, excitation spectra were not acquired. Furthermore, the evolution of a complex mixture of states precluded the assumption of a discrete, readily discernible number of distinct spectral contributions. We therefore adopted a more general strategy consisting of two stages, the first of which (Stage 1, below) reduces the data to a mathematical form in a model-free manner, permitting noise-free calculations leading to more detailed molecular inferences regarding intermediate states in Stage 2 (later section).

- We performed global fitting with the NonlinearModelFit function of *Mathematica* of a selection of sequential *s*pectra (time points) with a linear combination of (generally 4) hyperbolic functions **f**[λ] (Eq. 1). The four-parameter normalized hyperbolic function (Eq. 1, *Mathematica* notation), modified so as to reject solutions with complex numbers, was empirically selected for **f**[*λ*], because it exhibits rapid and robust parameter convergence and can represent without discontinuities virtually any shape and position of a single peak:
  1 $$\begin{aligned} {\mathbf{h}}[\lambda \_,\alpha \_,\beta \_,\mu \_,\delta \_] & : = {\mathbf{Re}}\left[ {\frac{{{\mathbf{Exp}}[ - \alpha \sqrt {\delta^{2} + (\lambda - \mu )^{2} } + \beta (\lambda - \mu )\sqrt {\alpha^{2} - \beta^{2} ]} }}{{2\alpha \delta \, {\mathbf{BesselK}}\left[ {1,\sqrt {\alpha^{2} - \beta^{2} } \delta } \right]}}} \right] \hfill \\ {\mathbf{f}}[\lambda ] &\,\,= \frac{{{\mathbf{h}}[\lambda ,\alpha ,\beta ,\mu ,\delta ]}}{{\mathop \smallint \nolimits_{{\varvec{\lambda}1}}^{{\varvec{\lambda}2}} {\mathbf{h}}[\lambda ,\alpha ,\beta ,\mu ,\delta ]{\text{d}}\lambda }}. \hfill \\ \end{aligned}$$
  The set of arbitrary functions was deemed neither uniquely nor individually to correspond to bands constituting the underlying molecular or ensemble (in the case of intermediates) components. The sole condition imposed was they cumulatively, i.e. as a suitably weighted linear combination, reproduce the experimental data with high accuracy. The first fit was to the initial *n* (*n* = 1–6) time points so as to determine the parameters of **f1**–**f4** (see ESM) and extrapolating the respective weights (linear coefficients, ESM) to 0 time (time point 1) to achieve a mathematical representation of the normalized monomer emission spectrum (more formally *q*[*λ*], the spectral quantal emission distribution function), **sp1**[*λ*] (Fig. 5).
- Applying the same procedure to the last *n* (*n* = 1–6) time points, a second set of functions **f1**–**f8** was derived and used to construct the spectrum of the final time point (**sp140**) and thereby that of the fibrillar state, **sp3**[*λ*], assumed to be the predominant species at the end of the reaction. We accounted for residual monomer by utilizing the relation:
  2 $${\mathbf{sp}}{\mathbf{3}}[\lambda ] = \frac{{wx \,{\mathbf{spx}}[\lambda ] - \alpha w1 \,{\mathbf{sp}}{\mathbf{1}}[\lambda ]}}{1 - \alpha }\,{\text{and}}\,w3 = \frac{wx - \alpha w1 }{1 - \alpha },$$
  in which *α* is the mole fraction of the monomer and *x* is number of a particular spectrum corresponding to mixture if the two components (in our calculations *x* = 60–140 and *wx* the weighting factor of spectrum *x*). Fitting spectra 60–140 led to *α* = 0.02 at the end of the reaction, and thereby to expressions/values for **sp3**[*x*] and *w*3 (see ESM). The weight caculations were based on the invariance of the total AS monomer concentration (150 µM), and the requirement that *w*1·**sp1** and *w*3*·* **sp3** reproduce the measured end spectra accurately (ESM).
- It was anticipated that the monomer and fibril spectra would also be represented in the intermediate spectra, reflecting the (co)existence of monomer and fibril and any other species in the molecular population with similar (or dissimilar spectra). All 140 time points of the 7MFE data set were accurately reproduced with a linear combination of *w*1·**sp1** and *w*3·**sp3** (ESM), indicating that all molecular subspecies present throughout the reaction and contributing to a finite extent to the overall spectrum had individual spectra given by a given (but different) combination of those of the monomer and fibril. This interesting yet surprising result\will be interpreted below. The linear fit coefficients (**x1**, **x3**) provided a measure of fractional concentrations (Fig. 6). In the case of the 7MFE data, these quantities did not sum to unity at intermediate points in the reaction (Fig. 6a). Considering that the recorded fluorescence signal at a given wavelength *λ* is proportional (via an instrument constant) to the product of (a) absorption cross-section *σ* (or extinction coefficient *ε*), (b) emission quantum yield QY, and (c) *q*[*λ*], and assuming invariant absorption properties, we concluded that one or more intermediates arising during the reaction had a lower QY, thereby implying a need to consider variable weights, i.e. *w*1[*i*], *w*2[*i*], and *w*3[*i*]; one should note that at least some of the intermediates can have a higher QY than that of the two end states. In view of the rapid decrease in signal intensity at certain wavelengths from the outset of the reaction, we attributed the decreased QY to an intermediate species associated with the monomer spectrum **sp1**. A reduction in relative QY to 0.2 ± 0.08 (s.d. over 30 time points) was calculated, leading to the revised relative (concentration) distributions given in Fig. 6c and their absolute contributions to the spectrum intensity in Fig. 6b. This procedure does not exclude a contribution of **sp3** to the intermediate, as is demonstrated below. Figure 6d shows the fluorescence signals originating from the three components at the particular emission wavelength, of 540 nm. The time course of the calculated total signal (F540) reproduces the sharp initial drop in recorded intensity measured in parallel experiments with a microplate reader.

The data for 6MFC labeled AS were more complex in that a simple combination of **sp1** and **sp3** determined for that probe no longer provided good fits for the intermediate time points. Two additional spectral components **f9** and **f10** were required, thereby defining a time dependent intermediate population, representable formally as *w*2[*i*]·**sp2**[*λ*] = *w*9[*i*]·**f9**[*λ*] + *w*10[*i*]·**f10**[*λ*]. The fit was very good but the relative variation of the weights *w*9[*i*] and *w*10[*i*] indicated that in the case of this ESIPT probe, the sequence of intermediates had distinctive and distinguishable spectral properties represented in the mean values (spectra) of the instantaneous population distribution. A tentative resolution into two intermediate spectral components species is given in Fig. 7. It is notable that the contribution and time courses of the two spectral components **sp2a** and **sp2b** are quite different, implying that they correspond to different reaction intermediates. A further analysis of this and other MFC data will be presented elsewhere.

In the context of the SAS scheme introduced earlier (Fig. 1) and the reaction model outlined below, reaction *phases* and *interfaces* were assigned to distinct events as follows: (1) formation of low order associates (dimers and higher order) and colloidal structures; (2) formation and processing of *fuzzy*; (3) amyloid nucleation; (4) onset of fibrillation and depletion of intermediates; (5) midpoint of fibrillation; and (6) completion of fibrillation. In order to relate this spectral classification to specific reaction intermediates in Stage 2 of the spectral analysis it is necessary to introduce a reaction model (next section) based on the extensive literature for the kinetics of amyloid formation (see “Introduction”) and incorporating novel features inferred from the structural elements of the SAS scheme (Fig. 1).

### Aggregation reaction model

The AFM images from the previous study (Fauerbach et al. 2012) alluded to above and featured in Fig. 1 established the *fuzzy ball* (*f1*), a nanosphere with a height of ~ 4 nm, as the earliest perceptible structure in AS incubations at all temperatures. The *fbs* appear to arise as colloidal clusters of AS that recruit extended AS molecules, forming a thin superficial (~ 1 nm height) layer perceived as *fuzziness*. The *fbs* subsequently coalesce into distinctive supramolecular structures, the *acunas*. The latter form spontaneously at 4 °C and at sufficiently high protein concentration (> 300 µM), but do not proceed further in the aggregation reaction sequence. That is, no fibril formation is observed upon prolonged incubation at 4 °C. The arrested reaction mixture also lacks the intense green T* band of 6MFC fluorescence characteristic of *mafs* (Fig. 5) and a ThioT signal is absent, implying the lack of secondary structure with β-sheets. Thus, these species are classified as pre-amyloid. Segmented *fuzzy fibers* are spawned and released from the *acunas* at higher temperatures (we have explored 37–70 °C), become denuded (change in segmentation frequency and loss of *fuzzy* coating), and finally evolve to *mafs*, differentiated by morphological features, such as height, pitch and length.

The above attributes led us to propose in this communication a new reaction scheme accounting for the kinetics of AS aggregation, one with general features that may also apply to other amyloid proteins (Fig. 8). A key novel element is the attribution of a catalytic role to colloidal AS in the formation and release of *fuzzy*, regarded as an obligatory precursor of amyloid nuclei with the incipient cross-β-sheet structure required for progressive fibrillation. Thus, up to three catalytic processes, two of them in novel cascade, and involving only a single protein, AS, without the influence of external agents, are incorporated in the scheme. The definitions and values of kinetic constants, the differential equations, and representative solutions yielding the time course of all the molecular entities are featured in the ESM. A key feature is that the three constants *k*3, *k*5 (and/or *k*9) and *k*7 must have finite values in order for fibrillation to proceed at all. The classical “lag phase” and “sigmoidal” fibrillation arise as natural consequences of the model, i.e. lack an inherent identity.

### Component analysis of the acquired spectra: Stage 2

We note that in Stage 1 the calculations were model independent, involving only reasonable assumptions about the nature of the initial (monomer) and end (fibril) species. We now seek to match the spectral analysis of Stage 1 with simulations conducted with different sets of kinetic parameters for the detailed scheme of Fig. 8. These were optimized iteratively and together with a matching matrix of relative (to momomer or fibril) quantum yields *s*[*k*, *i*], so as to yield approximations to the experimental spectra throughout the reaction time course to an acceptable degree of correspondence. The mathematical procedure was based on the linear relationship (Eq. 3) between the spectrum acquired at time point *i* and wavelength *λ* and the combination (product) of the spectral information derived from experiment in Stage 1, and the temporal concentration profiles generated by the solutions of simulations:

3 $${\text{Spectrum}}[i,\lambda ] = \mathop \sum \limits_{k = 1}^{{2\,{\text{or}}\,4}} w[k]{\mathbf{sp}}[k,\lambda ]\mathop \sum \limits_{{\varvec{j} = 1}}^{ \le 6} s[k,j] c[j,i],$$

where *k* is an index for the identified spectral components obtained in the Stage 1 analysis (1,2: **sp1**, **sp3**, respectively for both probes; 3,4: **sp2a**, **sp2b**, respectively, for probe 6MFC). The corresponding weights *w*[*k*] for **sp2a** and **sp2**b, were assigned arbitrarily and further incorporated into *s*[*k*,*j*]; *c*[*j*,*i*] the fractional concentration (in monomer units) of molecular component *j* at time point *i* according to the simulation (*j* ≤ 6); and *s*[*k*,*j*] dimensionless factors (generally 0, 1 or in the range 0–1) established directly for the start and end states or determined empirically (see below) so as to optimize the match between simulation and experiment. For each *s* matrix, we calculated the *molecular spectra*, **msp**[*j*,*λ*] corresponding to specific species represented in the reaction scheme using Eq. (4):

4 $${\mathbf{msp}}[j,\lambda ] = \mathop \sum \limits_{k = 1}^{{2\,{\text{or}}\,4}} s[k,j] {\mathbf{w}}[k] {\mathbf{sp}}[k,\lambda ],$$

**Fig. 9:**
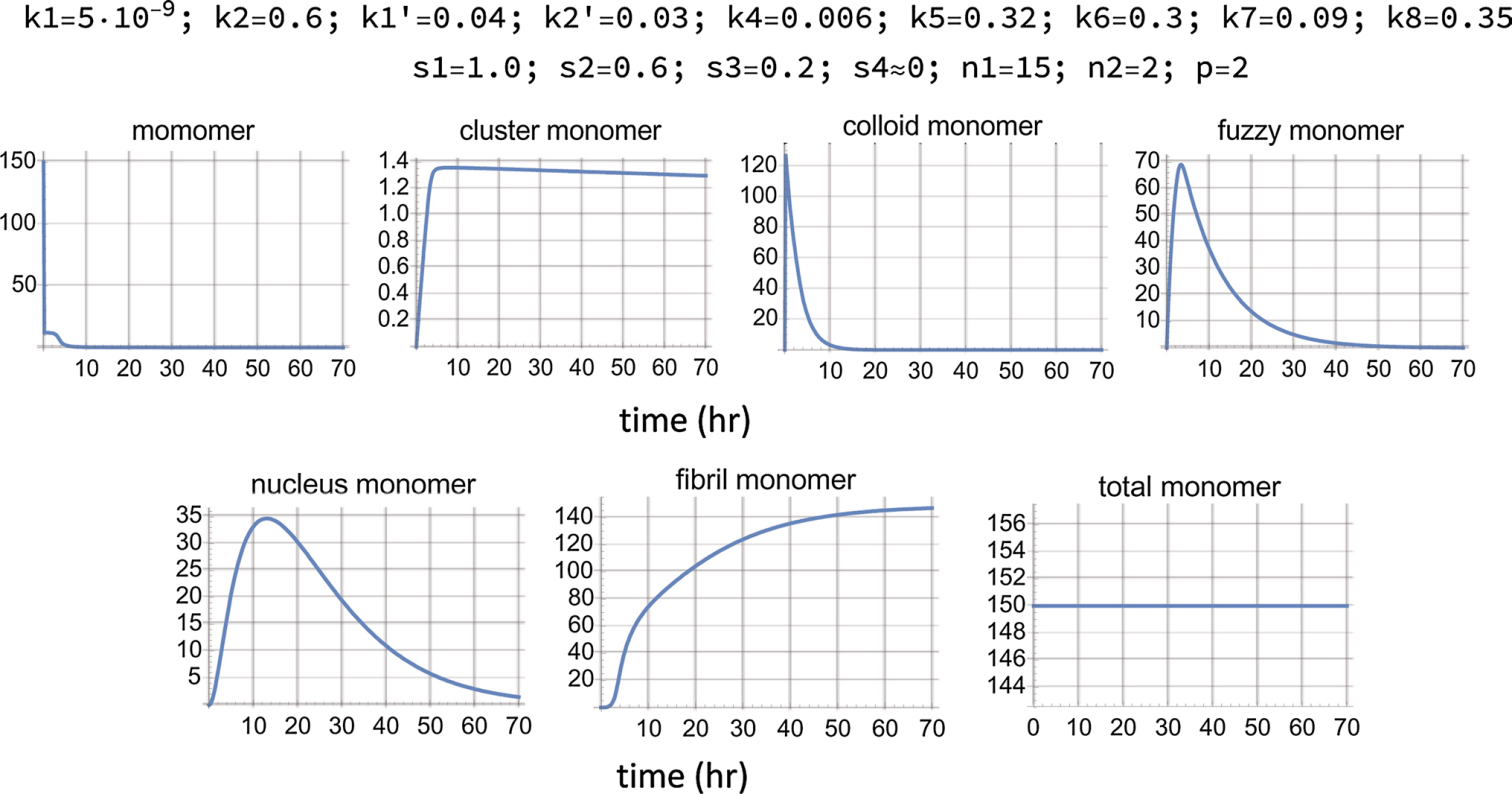
Solution of the set of differential equations embodying the reaction scheme of Fig. 8. The given set of parameters was selected for fitting the spectral components of the aggregation reaction probed with 7MFE, derived in the Stage 1 analysis and shown in Fig. 6. The last panel confirms the invariance of the total monomer concentration. The equations are given in the ESM. The values in this simulation of the stoichiometry parameters *n*1, *n*2, and *p* (defined in the ESM) were 10, 2, 2, respectively. The value of *k*8 is compatible with estimations of *k*_agg_ in the literature. The applicable units for the parameters are in µM and h

**Fig. 10:**
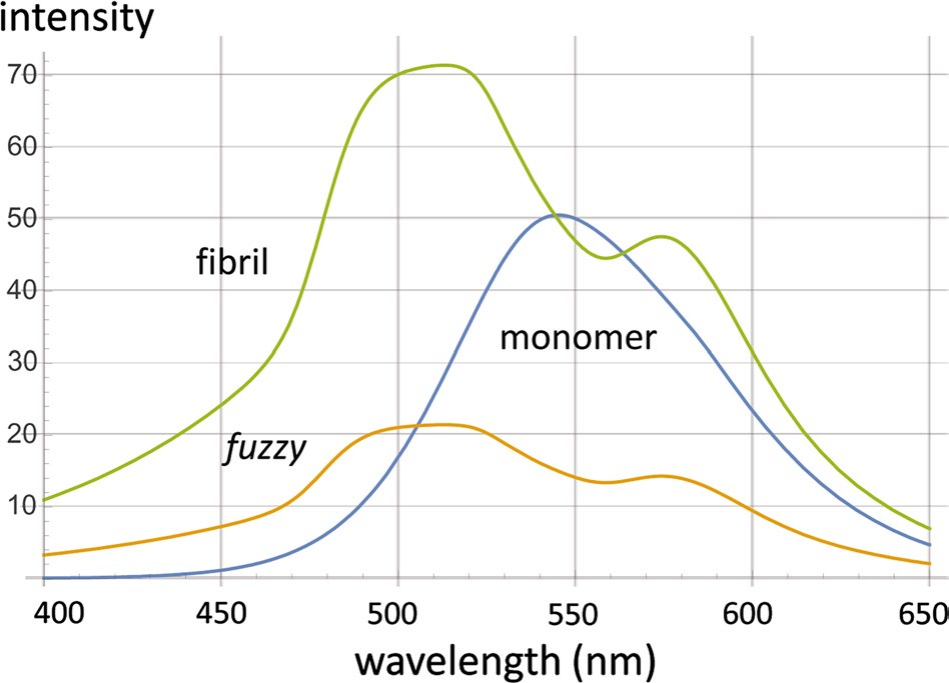
Molecular spectra computed for three major constituents of the aggregation reaction pathway of A18C-AS conjugated to 7MFE. See “Discussion” for interpretation of the protein microenvironment based on the properties of the ESIPT probe

Utilizing Eq. (4), kinetic simulations were conducted and a tentatively optimized (although most probably not unique) parameter set was identified (Fig. 9) from which it is inferred that **sp2** corresponds primarily to the *fuzzy* compartment of the reaction scheme of Fig. 8. The derived molecular spectra of (monomer, *fuzzy*, fibril) scaled by their relative signals at unit concentration are given in Fig. 10 and the relevant spectral parameters in Table 1. Their interpretation in terms of the corresponding protein structures and microenvironments are presented in the ensuing “Discussion”.

**Table 1:** ESIPT-labeled AS—spectroscopic parameters of the given molecular species

Probe	Molecular species	Number of bands	Position of bands (nm)	T*/N*	Intensities (A.U.)	Band assignment
7MFE	Monomer	1	545	n.a.	50	NH*
	*Fuzzy*	2	521, 574	1.3_4_	43, 32	N*, T*
	*Mafs*	2	513, 574	1.4_5_	70, 48	N*, T*
6MFC	Monomer	2	420, 518	0.70	60, 44	N*, T*
	*Mafs*	2	421, 542	1.9_4_	350, 680	N*, T*

**Fig. 11:**
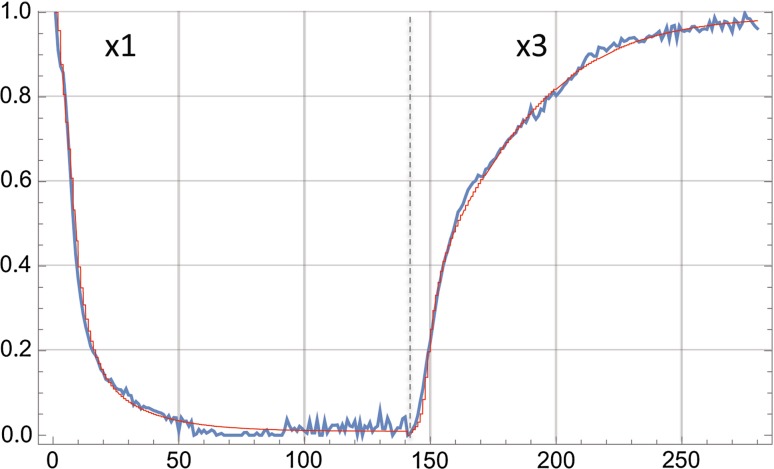
Fit (red) to the coefficient matrices **x1**, **x3** (blue) defining the contributions of **sp1** and **sp3** to the reaction spectra of 6MFE. The two data sets were catenated into a vector with a length of 280 (abscissa) and fit to the expression given by Eq. (5)

Although Eq. (3) is formally correct, its use involves an additional, redundant (after Stage 1) step of spectral fitting requiring data evaluations involving > 8000 data points and lacking an objective means for parameter optimization. However, the observation in Stage 1 that all spectra of the 7MFE probe could be represented as weighted combinations of **sp1** and **sp3** suggested a much simpler, powerful procedure based on nonlinear regression of the more restricted (2·140 points) coefficient data set (**x1**, **x3**) (Fig. 6a) coupled to solutions of the differential equations representing the kinetic scheme of Fig. 8. This can be achieved in *Mathematica* by incorporating into *NonlinearModelFit*, a function estimating **x1** and **x3** simultaneously in terms of the $s[k,j]\,{\text{and}}$ concentrations updated dynamically and iteratively within the module *ParametricNDSolveValue*, which provides solutions of differential–algebraic equations. The approach yielded a set of 14 iterated parameters that successfully (by visual inspection and regression index *R* ^2^) reproduced **x1** and **x3** and thus the entire time course of the experimental spectra (Fig. 11):

5 $$\{ \varvec{x}{\mathbf{1}}[i],\varvec{x}{\mathbf{3}}[i]\} = ind({\text{monomer}}[t] + s1 ({\text{clusters}}[t] + colloid[t]) + s2\, fuzzy[t]) + (1 - ind)\cdot(s3\, fuzzy\left[ t \right] + s4\, {\text{nucleus}}[t] + fibrils[t]),$$

in which *ind* (values 1,0) directs the fit to the **x1** or **x3** data, respectively; time *t* corresponds to spectral time point *i*; and the assumptions are made that only monomers, clusters, *colloid* and *fuzzy* contribute to the **sp1** spectral component and only *fuzzy*, nucleus and *fibrils* to **sp3**. The *s*[*k,j*] matrix (Eq. 3) is thus reduced to the relative quantum yields *s*1–*s*4 according to Eq. (5).

## Discussion

In this study we exploited specialized fluorescent probes and analysis methods to elucidate the initial self-assembly and (mis)folding stages of molecular α-synuclein. The novel reactive ESIPT probe, 7-MFE, was introduced at position 18 of AS to achieve enhanced environmental sensitivity towards the earliest stages of the monomeric AS folding and self-assembly process induced upon exposure to 37 °C. A new kinetic scheme was devised and utilized to interpret the experimental spectral data in in the context of the time course of specific molecular states of AS defined by a series of equilibria and kinetic pathways and their respective parameters. A key and novel element of the model is the proposal that colloidal entities arising by accretion of AS monomers provide an obligatory catalytic surface leading to the formation of an otherwise kinetically inaccessible conformational state, *fuzzy*, which progresses to the amyloid formation and propagation mechanisms via spontaneous conformational transitions. *Fuzzy* is also deemed to populate and promote the emergence of supramolecular species by colloidal association, thereby facilitating the formation of observed pre-amyloid fibrillar structures. The model also allows for a parallel pathway to nuclei via AS clusters and in this case, *fuzzy* assumes a catalytic role. We note that a templating “prion” mechanism (Eigen 1996) is not invoked explicitly. Our kinetic model reduces to most published schemes by judicious selection of parameter values.

### Interpretation of the ESIPT molecular spectra

The reaction scheme in Fig. 8 comprises six molecular species of AS: monomer, *colloid*, cluster, *fuzzy*, nucleus, and *fibrils*. As already indicated, *colloid* and nucleus generally appear at low concentrations and thus do not contribute appreciably to the observed emission spectrum, although finite levels of *colloid* are essential for the reaction mechanisms to function at all. The influence of optional clusters in the new model has not yet been extensively explored. However, in the case of the 7MFE probe, all three major species (monomer, *fuzzy* and *fibrils*) were successfully assigned distinctive emission spectra.

As in the case of 6MFC at position 140 (Yushchenko et al. 2010), the intensity of both bands increased at the end of the reaction and reached a high T*/N* ratio, indicative of a final environment of low polarity compared to the initial highly polar and protic state. 7MFE complements the fluorescent information provided by 6MFC by virtue of its sensitivity to H-bonding (Kenfack et al. 2012). The molecular spectrum for the monomer (**sp1**, Fig. 5) has a single band in the case of 7MFE and dual-band in the case of 6MFC (Table 1). The shape, position and number of bands for both ESIPT probes are similar to those observed for the unconjugated fluorescent probes in water (T*/N* ratio < 1 for 6MFC and a single band for 7MFE). These features suggest that the N-terminal of monomeric AS is in a highly polar environment; the probe located at position 18 is involved in intermolecular H-bonding with either a neighboring amino acid and/or with water. However, the total emission intensities (and therefore quantum yields) are significantly higher than in water. We conclude that covalent conjugation of the ESIPT probes to monomeric AS-A18C enhances probe solubility in water and provides a (partially) protected environment that block non-radiative decay pathways. This finding further supports the experimental evidence for an ensemble of partially folded conformations of AS under physiological conditions.

The molecular spectra of *mafs* (**sp3**, Fig. 5), of dual-band character in both cases, represents an end-state of reduced polarity compared to the monomer (**sp1**). In the case of 6MFC, the positions of the peaks change only slightly between **sp1** and **sp3** (Table 1). However, the absolute intensities of both N* and T* bands increase significantly albeit asymmetrically such that T*/N* ratio went from ~ 0.7 for **sp1** to ~ 1.9 for **sp3** (Table 1). A more dramatic change was observed with 7MFE, for which the initial single-band NH* band transformed into a dual-band spectra with maxima at ~ 513 and 574 nm and a T*/N* ratio of ~ 1.5. In the molecular spectra for **sp3**, the central NH* no longer predominated The end-state spectrum of 7MFE is similar to that of 3HF compounds in ethyl acetate with 3% of water as a co-solvent (Shynkar et al. 2004), suggesting that a significant loss of hydration occurs, a phenomenon perceived only due to the H-bonding sensitivity of 7MFE at position 18 of AS. As in the case of 6MFC, the polarity revealed in **sp3** was also lower than that inferred from **sp1,** reflecting the progressive formation and/or restructuring of binding sites involving the N-terminus in molecules engaging cross-β-sheet interaction, such as in *mafs*.

In combination with **sp1** and **sp3**, the molecular spectrum **sp2** of 7MFE assigned to the *fuzzy* species accommodates all the acquired spectra in the time series. The *fuzzy* spectrum shares features of both the monomer and fibril species. The transition from the monomer to the intermediate species occurs rapidly, as evidenced by a loss of fluorescence intensity at certain wavelengths (Fig. 6D), Such a spectral response may reflect an increase in the contribution of non-radiative decay due to greater hydration and/or higher solvent exposure. We conclude that *fuzzy* exhibits significant secondary structure that probably undergoes significant transitions on the path to amyloid nuclei, i.e. pre-amyloid-to-amyloid transformation depicted in the reaction scheme (Fig. 8) and discussed further below. Although the structural inferences from such experiments are necessarily limited, the ability to monitor the time course of reaction from start to finish constitutes a significant advance over the capabilities of more limited yet universally employed amyloid probes such as ThioT.

### Role of colloidal structures in catalytic formation of *fuzzy*

The formation of colloidal nanoparticles (“colloid bodies”) is an early step, probably universal to all amyloid-forming systems (Anson et al. 2013; Woodard et al. 2014). One perceives such entities in published images albeit without explicit recognition as a significant participant in the on-pathway mechanism. Emphasis in the literature is generally placed on AS “oligomers”, the associated protein states recovered by physical isolation (see “Introduction” for references); in our model (Fig. 8) oligomers = clusters + *fuzzy.* A major asset of the fluorescence assay employed in this study is that high, multiparametric selectivity can be achieved by continuous monitoring of solutions, avoiding the perturbation introduced by an isolation procedure. The oligomers are classified in the literature (see “Introduction”) according to the attributes of time of apparition, backbone redistribution and acquisition of secondary and tertiary structure, and biological (more generally, biochemical) activity. Early oligomers are generally deemed to be relatively extended, polymorphic, stabilized by hydrophobic interactions and involvement of anti-parallel β-structure, and a modest degree of association (e.g., 2–32) (Ahmad et al. 2012; Celej et al. 2012; Cerf et al. 2009, Gallea and Celej 2014, Neupane et al. 2014; Paslawski et al. 2014; Rekas et al. 2010; Sierecki et al. 2016; Zijlstra et al. 2012). Of particular interest to our study is the observation that intramolecular diffusion precedes bimolecular association and that the N-terminus dynamics are slowed by interactions at a distance (Ahmad et al. 2012), but also locally (Gallea and Celej 2014). Late oligomers are more compact. Because of the diversity of criteria and experimental protocols, the early-late assignment of toxicity in the literature (Krishnan et al. 2012; Pieri et al. 2016) is somewhat ambiguous,^2^ and we do not assign this property to particular member(s) of our kinetic scheme. However, spectroscopically defined *fuzzy* appears to correspond most closely to the class of early oligomers in view of its time course, the ESIPT-probe derived inferences featured in the previous section, and the central role(s) we have assigned to this component in the kinetic scheme.

Following our introduction of *fuzziness* in 2012, a very interesting account appeared of a presumably related structure, denoted “fuzzy coat”, observed by transmission EM and AFM microscopy of pathological human Tau fibrils (Wegmann et al. 2013). The ~ 16 nm thick coat comprises the unstructured C-terminal and N-terminal domains of stacked TauRD constructs. The mechanical (stiffness) and adhesive properties are modulated by salt and pH, features that the authors propose regulate the formation and (de)stabilization of neurofibrils and tangles, and soluble, toxic, oligomers in the cellular context.

The postulate that *fuzzy* originates on the surface of a colloidal form of AS requires an exposition of the fundamental requirements for forming and stabilizing such structures. A colloid is typically a two-phase system consisting of a continuous phase (the dispersion medium, in our case water) and a dispersed phase comprising the particles that generally lie in the range 1–100 nm/1 µm. The ratio of surface area to volume decreases with particle size and thus the size depends on the factors providing stabilization against aggregation and/or phase separation. The two main mechanisms for colloid stabilization involve steric and electrostatic modifications. Surface charge, naturally occurring or added, increases electrostatic stability and phase separation, while adsorption of polymers enhances stability sterically.

We propose that the AS nano-colloids achieve a size dictated by the net (negative) molecular and collective charge. The negatively charged surface attracts free AS molecules, which bind selectively via their positively charged N terminus, providing the electrostatic stabilization and particle growth limitation alluded to above. The dense layer constitutes the *fuzziness* perceived by AFM and constitutes a form of “polymer brush”, a construct that is well understood and widely applied (Azzaroni 2012). Gold and other nanoparticles such as quantum dots provide platforms for the facilitated nucleation of AS aggregation (Alvarez et al. 2013; Joshi et al. 2015; Lin et al. 2017; Roberti et al. 2009) although the surface effects lead to a complex set of influences, some nonintuitive such as the acceleration of fibril formation by *strong* attractive forces (Vacha et al. 2014).

We suggest further that closely packed AS molecules constituting the *fuzzy* layer of the *fbs* engage in local interactions, including the formation of intermolecular anti-parallel β-sheets or interacting helical regions (Sahu et al. 2015) defining and stabilizing a form of dimer. In previous studies of the acceleration of fibrillation by polyamines (Fernandez et al. 2004) and AS-coated quantum dots (Roberti et al. 2009) we inferred that AS dimers coupled to the release of long-range interactions in the monomer (Bertoncini et al. 2005) facilitate nucleation. We note that a parallel orientation of the AS molecules and tethering via the N-terminus would be favored this process, judging from the relative bimolecular rate constants for protein–protein dimerization of the single cysteine mutants in a two-step reaction mediated by 5,5′-dithiobis-(2-nitrobenzoic acid) (DTNB) (NejatyJahromy 2006). Dimerization at position 18 is 30 times faster than at position 140. The non-covalent dimers would be released as such into the *fuzzy* pool (*p* = 2 in the scheme of Fig. 8), undergoing further concerted association and rearrangements leading to the parallel β-sheet element of the amyloid nucleus in Fig. 8. One can speculate further that this mechanism passes through a tetrameric intermediate (dimer of dimers) (Dettmer et al. 2015) with a subsequent resolution into to 2 dimers with the alternative secondary structure. AS–AS interactions *between fbs* would also trigger the creation of the supramolecular family (Fig. 1), including the *acuna*. It is remarkable that both stabilizing and destabilizing influences mediated by a single protein can give rise to the complex set of reactions represented only partially by the scheme of Fig. 8. Of therapeutic interest is the possibly for targeting the key loci and players in the mechanism, such as by neutralizing *fb* catalysis or trapping *fuzzy* subpopulations.

As a concluding remark we would point out that the experimental and analytical tools illustrated in this report can be refined so as to enable a more detailed view of the entire aggregation reaction pathway using single or a combination of probes, which are obviously not restricted to the ESIPT class. More specificity for the reaction intermediates and improved spectroscopy modalities (fast arrays, coupled excitation and emission recording, anisotropy and lifetime measurements) coupled with advanced data analysis are required but readily available.

## Electronic supplementary material

Below is the link to the electronic supplementary material.

Supplementary material 1 (PDF 1054 kb)

## Abbreviations

AS: α-Synuclein
wtAS: Wild-type AS
ESIPT: Excited-state intramolecular proton transfer

## Footnotes

1 Aggregation is a term more appropriate for a process occurring on the macroscale, e.g. as a consequence of mechanical stirring in the laboratory environment. In the cell, the localized, individual elongation of fibrils leads to entanglement in a matrix. Amorphous aggregation is an alternative, often coexistent, physical phenomenon also exhibited by amyloid proteins under certain solution conditions.

2 A citation from the literature is perhaps appropriate: “Our findings reveal the inherent multiplicity of the process of protein misfolding and the key role the β-sheet geometry acquired in the early stages of the self-assembly process plays in dictating the kinetic stability and the pathological nature of individual oligomeric species.” (Chen et al. 2015).
