# Supplementary material for: Pre-aggregation kinetics and intermediates of α-synuclein monitored by the ESIPT probe 7MFE
Source: Eur Biophys J. 2017 Dec 18;47(4):345–62. doi: 10.1007/s00249-017-1272-0 (PMC5982440; doi:10.1007/s00249-017-1272-0)
Supplement: Supplementary file 1 — Supplementary material 1 (PDF 1054 kb) [file 249_2017_1272_MOESM1_ESM.pdf]

Supplementary information

Pre-aggregation kinetics and intermediates of alpha-synuclein monitored by the ESIPT probe 7FME

Jonathan A. Fauerbach and Thomas M. Jovin

Table of contents:

- ***AS expression and purification***
- **Synthesis of 6MFC**
- **Synthesis of 7MFE**
- ***AS fluorescent labeling***
- ***AS aggregation***
- ***Spectral deconvolution***
- ***AS species analysis***
- ***Kinetic modeling***

1. ***AS expression and purification (wt and mono-cysteine mutants)***
Protocols were performed as described in (Fauerbach, Yushchenko et al., 2012). Wildtype human α-synuclein (wtAS) and monocysteine mutants were expressed in *E. coli* BL21 (DE3) cells. The production of the recombinant protein was according to a 2009-2010 protocol (currently being revised) of the Laboratory of Cellular Dynamics (MPIbpc). Transformation was with pT7-7 plasmids encoding wildtype and mutant protein sequences. Expression was initiated by inoculation of a volume of 900 ml LB medium with 100 ml of a pre-culture and addition of 1 ml of 100 mg/ml ampicilin. Incubation was carried out at 37 °C with agitation (400 rpm) until the A600 reached a value of 0.5-0.8. Expression of the recombinant protein was induced by addition of IPTG and incubation was continued for an additional 4-6 h. The bacterial cells were harvested by centrifugation (Beckman JA10 rotor, 5000 rpm, 30 min 4 °C) and the pellets were stored at -20 °C. Cell pellets were thawed with 15- 20 ml lysis buffer containing 10 mM Tris-HCl, pH 7.7, 1 mM EDTA and 1 mM protease inhibitor PMSF. In the case of cysteine-containing mutants, 5 mM dithiothreitol (DTT) was added. Three freeze-thaw cycles were performed with liquid N2 and 70 °C hot water. The cells were lysed by sonication using a Fisher Scientific Digital Sonic Dismembrator Model 500 with a 6.5 mm diameter Branson microtip. Cells were subjected to three 10 s periods of sonication at 50% maximum amplitude, with 10 s intervals on ice. The extract was centrifuged 15 min at 2500×g and the supernatant was recovered. Proteins other than AS were denatured/precipitated by boiling at 100 °C for 20 min followed by centrifugation at 37000×g for 30 min at 4 °C. DNA was removed by addition of streptomycin sulfate to the supernatant to a final concentration of 10 mg/ml, agitation of the solution for 15 min at 4 °C, and centrifugation for 30 min at 4 °C and 37000×g. AS was precipitated from the supernatant by addition of solid $(NH_4)_2SO_4$ to a final concentration of 360 mg/ml and subsequent centrifugation as above. The pellets containing AS were stored at -20 °C. Purification of AS was carried out with a GE Healthcare ÄKTA explorer FPLC system equipped with an Applied Biosystems POROS HQ 20 µm, 4.6x100 mm (1.7 ml) column. Samples were concentrated with Millipore Amicon 10 kDa filters and ultracentrifuged for 1 hour at 105 ´g in a Beckman 80 TLS-55 rotor. The supernatant was recovered, diluted to a convenient concentration and frozen with liquid N2, with storage at -80 ºC.

2. ***Synthesis of 6MFC. 6***MFC was synthesized as described in th SI of (Yushchenko, Fauerbach et al., 2010).

3. ***Synthesis of 7MFE.*** 7MFE was synthesized according to the PhD thesis of J. Fauerbach: (http://digital.bl.fcen.uba.ar/Download/Tesis/Tesis_5357_Fauerbach.pdf). A summary is found below:

The following information describes in detail the steps involved in the synthesis of 7MFE, starting from N-(3-hydroxyphenyl)acetamide (25). The objective is to obtain between 1-10 mg of 7-MFE.

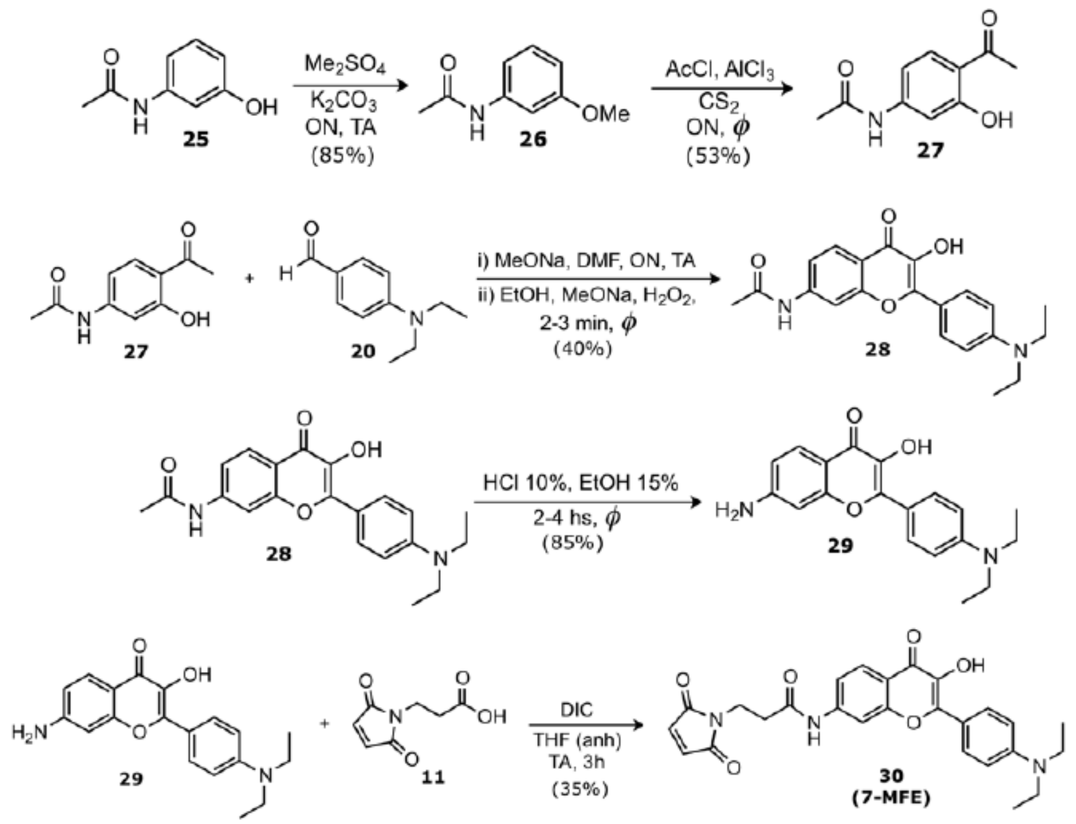

Step 1. Synthesis of N-(3-methoxyphenyl)acetamide (26).

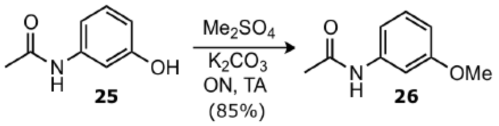

Procedure. Take a 250 ml round flask and add 53 g of dry K_2CO_3 (384 mmol) and a stirring bar. Then place flask in an oil or sand-bath, connect a reflux condenser and purge the system with Argon. Then add 50 ml of dry acetone and mix vigorously, creating a suspension. Add 10.0 g (66.2 mmol) of N-(3-hydroxyphenyl)acetamide (25) and 19 ml of methyl sulfate (200 mmol, d=1.33 g/ml). Both are available from Sigma Aldrich. After that, the system is heated and allowed to reflux for 16 h. The following day, heating is turned off and the reaction is allowed to cool to RT. The suspension is the filtered and washed with acetone. The flow-through is tested after each wash by depositing a drop on a TLC silica gel plate and examining under UV. The solid is washed until no visible spot appears under UV. The acetone solution is saved and the solid discarded. The excess acetone is evaporated in a rotary evaporator. As acetone is removed and the solution becomes concentrated, a white precipitate becomes visible. Acetone is not completely removed, and enough is added so as to redissolve the white precipitate. A 5% solution of NH_3 in water is added slowly to basify and help destroy any leftover methyl sulfate. DCM or chloroform are then used to extract the desired compound from the aqueous basic solution. The organic phase is saved, dried with sodium sulfate (anh.), filtered and evaporated.

Purification. The desired compound is furthered purified by 'dry flash' column chromatography using cyclohexane/ethyl acetate (from 99:1 to 95:5), yielding 9.3 g (56.3 mmol, 85%) of product 26.

1H NMR (200 MHz, CDCl3) d (ppm) 8.1 (s, 1H, -NH), 7.73 (t, J = 1.29 Hz, 1H, H4), 7.42-7.28 (m, 1H, H-3,5), 6.67 (dt, J = 7.3, 1.3 Hz, 1H, H-1), 3.7 (s, 3H, H12), 2.06 (s, 3H, H-11).

Step 2. Synthesis of 4’-acetamide-2’-hydroxyacetophenone (27).

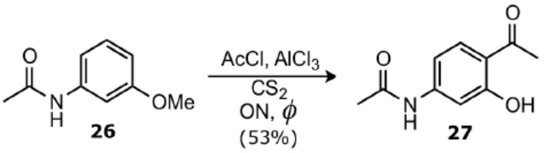

Procedure. Add 30 ml of dried carbon disulfide (CS_2) in a round flask. Place the flask in a water/ice bath and allow 10-15 min to equilibrate. Add 4.0 g (24.3 mmol) of compound 26 (obtained from step 1). Mix vigorously. Let the suspension cool, then, add 5.0 ml (70 mmol) of acyl chloride, followed by aluminum trichloride (12 g, 90 mmol). $AlCl_3$ should be added in 4-5 small portions over a period of 30 min so that the temperature does not rise above 35-40 °C after each addition. When all $AlCl_3$ has been added, the reaction is allowed to reach RT over an hour and then heated until reflux over 3 h. After this reflux time, the reaction is cooled to RT and then poured into water/ice where the product precipitates.

Purification. After 10-15min, the water suspension is filtered and purified by ‘dry flash’ column chromatography using cyclohexane/ethyl acetate (from 99:1 to 80:20), yielding 2.49 g (12.9 mmol, 53%) of product 27.

1H NMR (301 MHz, DMSO-d6) d 12.31 (s, 1H, -OH), 10.21 (s, 1H, -NH), 7.80 (d, J = 8.8 Hz, 1H, H-6), 7.33 (d, J = 1.8 Hz, 1H, H-3), 7.05 (dd, J = 8.8, 1.9 Hz, 1H, H-1), 2.55 (s, 3H, H-12), 2.07 (s, 3H, H-10). 13C NMR (126 MHz, DMSO-d6) d 202.9 (C-8), 169.1 (C-11), 162.5 (C-4), 146.2 (C2), 132.3 (C-6), 115.3 (C-5), 109.9 (C-1), 105.7 (C-3), 26.6 (C-12), 24.2 (C-10).

MS (ESI) calculated for C10H11NO3 (M+H)+: 194.0817, found: 194.0813 (+mode). (M+Na)+: 216.0637, found: 216.0632 (+mode). (M-H)-: 192.0661, found: 192.0665 (-mode).

Step 3. Synth of 7-acetamide-2-(4’-diethylaminophenyl)-3-hydroxychromone (28).

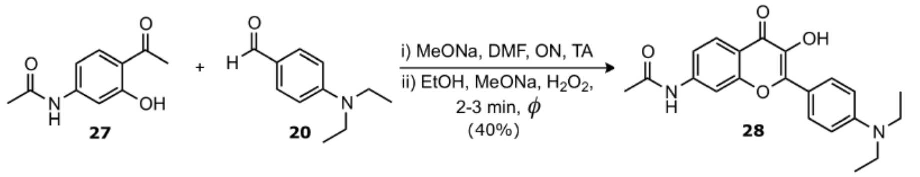

Procedure. In a 1 L round flask add 752 mg (3.89 mmol) of compound 27 (obtained in step 2) and 750 mg (4.24 mmol) of 4-(dimethylamino)benzaldehyde (20), avaikble from Sigma Aldrich, and 10 ml of dry DMF. Dissolve everything. Add 750 mg (13.89 mmol) of sodium methoxide and leave stirring ON at RT. The next day, prepare a heating mantle and a bucket full of ice. It is important to have this ready before starting by adding 30 ml of ethanol to dilute the reaction mixture and facilitate boiling more. Briefly mix well, then, add 5 g (92.6 mmol) of sodium methoxide. Briefly mix the suspension. Finally add all at once 7 ml of 30% hydrogen peroxide. The solution should start to heat up and boil almost immediately. If it doesn’t , briefly place it on a pre-heated mantle to help the flask reach the appropriate temperature. When the reaction mixture boils (by observation of bubbles and/or condensation of ethanol on the side or top of the flask), remove from mantle. The reaction mixture should be able to self-sustain the temperature. Allow it to boil for only 3 min (no more than 5 min). Then place and bury flask on ice and cool it down for ~15 min. Finally pour the reaction mixture on ice/water in a 500 ml flask or beaker provided with a stirring bar. Neutralize to pH 5-6 with HCl, checking with pH-paper strips. A color change should be evident in the solution while neutralizing. At a pH of 5-6, the solution becomes a brown fine suspension. If the pH drops below the indicated limit, the suspension changes again into a solution. Regardless the pH, when a very fine suspension is noticeable stop and filter using an appropriate paper (for fine solid)s.

Purification. The desired compound is purified by normal column chromatography using cyclohexane/ethyl acetate (from 9:1 to 7:3), yielding 568 mg (1.55 mmol, 40%) of an orange(-ish) product 28. Before purification, the product should be checked by TLC. A yellow-orange spot should be apparent, with fluorescence under long wavelength illumination (~365 nm).

1H NMR (301 MHz, DMSO-d6) d 10.41 (s, 1H, -NH), 8.89 (s, 1H, OH), 8.14 (s, 1H, H-3),8.05 (d, J = 8.9 Hz, 2H, H-17,19), 7.99 (d, J = 8.7 Hz, 1H, H-6), 7.40 (d, J = 8.6 Hz, 1H, H-1), 6.79 (d, J = 9.0 Hz, 2H, H-16,20), 3.41 (q, J = 6.8, 6.8, 6.8 Hz, 4H, H-22,23), 2.13 (s, 3H, H-14), 1.13 (t, J = 6.9, 6.9 Hz, 6H, H-24,25).

13C NMR (126 MHz, DMSO-d6) d 171.2 (C-10), 169.1 (C-15), 154.9 (C-4), 148.2 (C-18),146.5 (C-8), 143.2 (C-2), 136.7 (C-9), 128.9 (C-16,20), 125.2 (C-6), 117.1 (C-13), 116.7 (C-5), 115.8 (C-1), 110.8 (C-17,19), 105.7 (C-3), 43.6 (C-22,23), 24.1 (C-14), 12.4 (C-24,25).

MS (ESI) calculated for C21H22N2O4 (M+H)+: 367.1658, found: 367.1652 (+mode). (M-H)-: 365.1501, found: 365.1507 (-mode).

Step 4. Synthesis of 7-amino-2-(4'-diethylaminophenyl)-3- hydroxychromone (29).

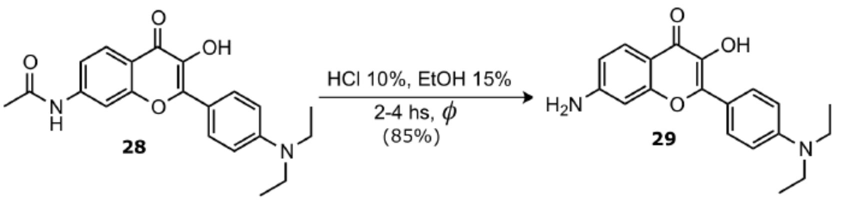

Procedure. In a round flask, add 200 mg (0.546 mmol) of product 28 (obtained in step 3) in 5 ml of 10% HCl (aq) with a 15% of ethanol. Heat to reflux for 3-5 h. Then add some water and neutralize to pH 6-7. An orange (fine) solid should precipitate. Filter, wash with small amount of water, and dry under vacuum ON. This can be use without further purification (checked by TLC and NMR), yielding 150 mg (0.462 mmol, 85%) of product 29. By TLC this should also fluoresce under long wavelength illumination.

Alternative procedure. Although the following procedure was not explicitly tested for these compounds, it was tested for amide isomer. If available, this reaction is possible under Microwave irradiation instead of heating reflux. Example: in a 10 ml microwave vial, 23 mg of the acetamide-chromone is suspended in 3 ml of 10% HCl plus 1 ml of 99% ethanol. The suspension is sonicated briefly to achieve maximum dispersion. The vial is sealed and heated to 140-150 °C for 15min with 500 rpm mixing (Anton Paar Monowave 300). Proceed to filter, wash with water and dry under vacuum ON. Yield: 85-95%

1H NMR (400 MHz, DMSO-d6) d 8.58 (s, 1H, -OH), 7.98 (d, J = 9.2 Hz, 2H, H-12,13), 7.72 (d, J = 8.6 Hz, 1H, H-3), 6.78 (d, J = 9.4 Hz, 2H, H-12,13), 6.65 (dd, J = 8.6, 2.0 Hz, 1H, H-5), 6.55 (d, J = 2.0 Hz, 1H, H-4), 6.21 (s, 2H, H-24), 3.41 (d, J = 7.0 Hz, 4H, H-20,21), 1.13 (t, J = 6.9 Hz, 6H, H-22,23).

13C NMR (101 MHz, DMSO-d6) d 171.54 (C-9), 157.21 (C-2), 154.20 (C-6), 148.33 (C-16), 144.93 (C-8), 136.36 (C-7), 129.05 (C-12,13), 126.23 (C-3), 118.09 (C-11), 113.45 (C-5), 111.57 (C-1), 111.22 (C-14,15), 97.84 (C-4), 44.12 (C-20,21), 12.91 (C-22,23).

MS (ESI) calculated for C19H20N2O3 (M+H)+: 325.1552, found: 325.1545 (+mode). (M-H)-: 323.1396, found: 323.1406 (-mode).

Step 5. Synthesis of 7-MFE (30).

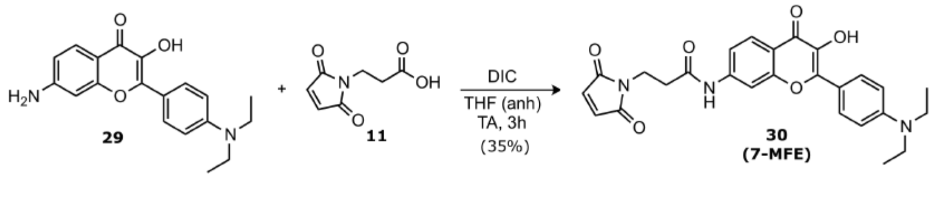

Procedure. In a round flask, dissolve 15 mg (0.046 mmol) of product 29 (obtained in step 4) in 5 ml anhydrous THF. Add 3-maleimidepropanoic acid (11, 7.8 mg, 0.047 mmol). Then add 500 µl of DIC (09) at RT. After 3h the reaction is filtered and carefully washed with DCM. Purification: the washed solid is purified by preparative TLC using cyclohexane/ethyl acetate (6:4) and revealed by both short and long UV wavelength. The scratch

band is then washed and product extracted with Ethyl Acetate, obtaining product 30 with a 35% yield (7.6 mg, 0.016 mmol).

1H NMR (500 MHz, DMSO-d6) d 10.54 (s, 1H, -NH), 9.02 (s, 1H, -OH), 8.12 (d, J = 1.9 Hz, 1H, H-4), 8.06 (d, J = 9.1 Hz, 2H, H-14,15), 7.99 (d, J = 8.7 Hz, 1H, H-3), 7.37 (dd, J =8.8, 1.9 Hz, 1H, H-5), 7.05 (s, 2H, H-32,33), 6.81 (d, J = 9.3 Hz, 2H, H-12,13), 3.76 (t, J= 7.0 Hz, 2H, H-28), 3.46 (s, 4H, H-20,21), 2.69 (t, J = 7.0 Hz, 2H, H-26), 1.15 (t, J = 7.0 Hz, 6H, H-22,23).

13C NMR (126 MHz, DMSO-d6) d 171.7 (C-9), 171.3 (C-30,31), 170.0 (C-25), 155.4 (C-2), 148.8 (C-16), 147.1 (C-8), 143.5 (C-6), 137.3 (C-7), 135.1 (C-32,33), 129.6 (C-14,15), 125.8 (C-3), 117.5 (C-11), 117.4 (C-1), 116.5 (C-5), 111.3 (C-12,13), 106.5 (C-4), 44.2 (C-20,21), 35.7 (C-26), 34.2 (C-28), 12.9 (C-22,23).

4. ***AS fluorescent labeling.*** Mono-cysteine mutants of AS were covalently labeled with MFC and 7MFE as described before in (Fauerbach et al., 2012). For the labeling procedure, the maleimide fluorophores (MFC & 7MFE) were dissolved in DMSO and mixed dropwise with a solution of ~300 µM of monocysteine AS (i.e. AS A18C, AS A140C) in 25 mM Na-PO_4 pH 7.3 with 3 mM tris(2-carboxyethyl)phosphine (TCEP); the final DMSO:buffer ratio was 1:2. MFC was in a 5-10-fold molar excess over the protein. The solution was left at 4 ºC under mixing overnight (ON). The next day the reaction mixture was diluted 10-fold with 25 mM Na- PO4 pH 7.3, 3 mM TCEP and concentrated by 3-4 passages through a Millipore Amicon 10 kDa filter to a volume of ~ 1 ml in order to remove DMSO from the reaction mixture. A size exclusion PD-10 column was used as a first step of purification from the unbound dye. A Pharmacia Smart chromatography system was used with a Superdex 200 column (10/300) and 25 mM Tris-HCl, pH 7.2, 150 mM NaCl elution buffer to separate thoroughly the excess reagent from the labeled protein and the unlabeled protein. The extent of labeling was 50-70%. Samples were frozen with liquid N_2 and stored at -80 ºC.

5. ***AS aggregation.*** Aggregation assays of wtAS protein in combination with cysteine-containing mutants labeled with the MFC ESIPT probe were performed at 37 °C, as described previously (Fauerbach et al., 2012, Yushchenko et al., 2010).

6. ***Spectral deconvolution.*** The procedure is described in the text. All programs and calculations were implemented in *Mathematica 11.1*. Global fits of a set of hyperbolic functions to the initial and final phases of the aggregation reaction served to define **sp1** and **sp3**. For example, the functions used for the 7MFE data were as follows.

hyperbolic function:

$$\texttt{f}[x\_, \alpha\_, \beta\_, \mu\_, \delta\_] := \texttt{Re}\left[\frac{e^{-\alpha\sqrt{\delta^2+(x-\mu)^2}+\beta(x-\mu)}\sqrt{\alpha^2-\beta^2}}{2\,\alpha\,\texttt{Abs}[\delta]\,\texttt{BesselK}\left[1, \sqrt{\alpha^2-\beta^2}\,\texttt{Abs}[\delta]\right]}\right]$$

for monomer spectrum:

```
f1[x_] := 1.0312662507551646` f[x, 0.09611168973263413`, 0.05692647147892009`, 560.3231901947959`, 25.74634560262209`]
f2[x_] := 1.2457171642251366` f[x, 0.13286603049158227`, -0.013601424875455098`, 517.8866277383512`, 110.03745901070839`]
f3[x_] := 1.0377132969000742` f[x, 0.18102273081632314`, -0.10362554344820042`, 576.4728049702911`, 53.445432511420734`]
f4[x_] := 1.0610857537105085` f[x, 0.03078110954406457`, -0.0031727558914268804`, 585.6127474356508`, 6.282145687325883`]
```

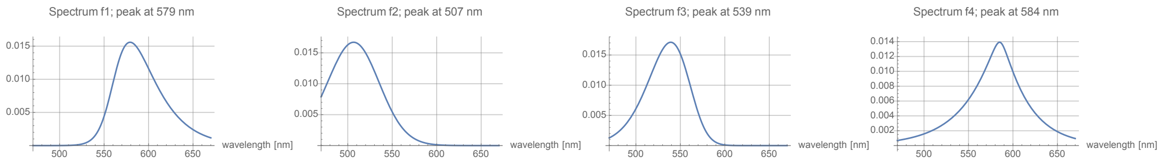

for fibril spectrum:

```
f5[x_] := 1.0682786900418946` f[x, 0.2960947679050569`, 0.25958699875338476`, 553.3276574258765`, 20.069531424949304`]
f6[x_] := 1.1013421357680686` f[x, 0.16402962096989498`, 0.11722825783668811`, 476.88206405581747`, 15.62848821970986`]
f7[x_] := 1.8244959454467762` f[x, 0.035073892752405675`, -0.02094245448335714`, 523.3064817751906`, 8.01277532920815`]
f8[x_] := 1.3388607578519678` f[x, 0.09169571965883148`, -0.07114595698445328`, 599.2328435256761`, 44.461247622100316`]
```

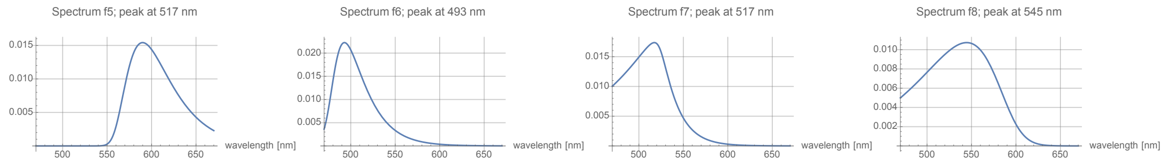

monomer (sp1) and fibril (sp3) spectral functions:

```
sp1[x_] := 0.34752750885077965` f1[x] + 0.5647486135520031` f3[x] + 0.0877238775978723` f4[x];
w1 = 4706;
sp140[x_] := 0.2125185769103166` f5[x] + 0.13409194987601816` f6[x] + 0.20815857977038715` f7[x] + 0.44523089336762856` f8[x];
w140 = 7558;
sp3[x_] := (w140 sp140[x] - 0.02 w1 sp1[x]) / (w140 - 0.02 w1);
w3 = (w140 - α w1) / (1 - α) /. α → 0.02 = 7616
```

fits to the extreme spectra (time points 1, 140):

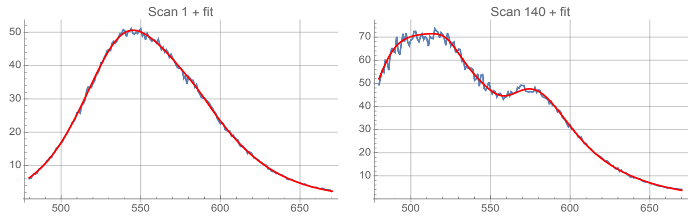

representative fits to the entire data set, individually with each spectrum, using linear combinations of sp1 and sp3:

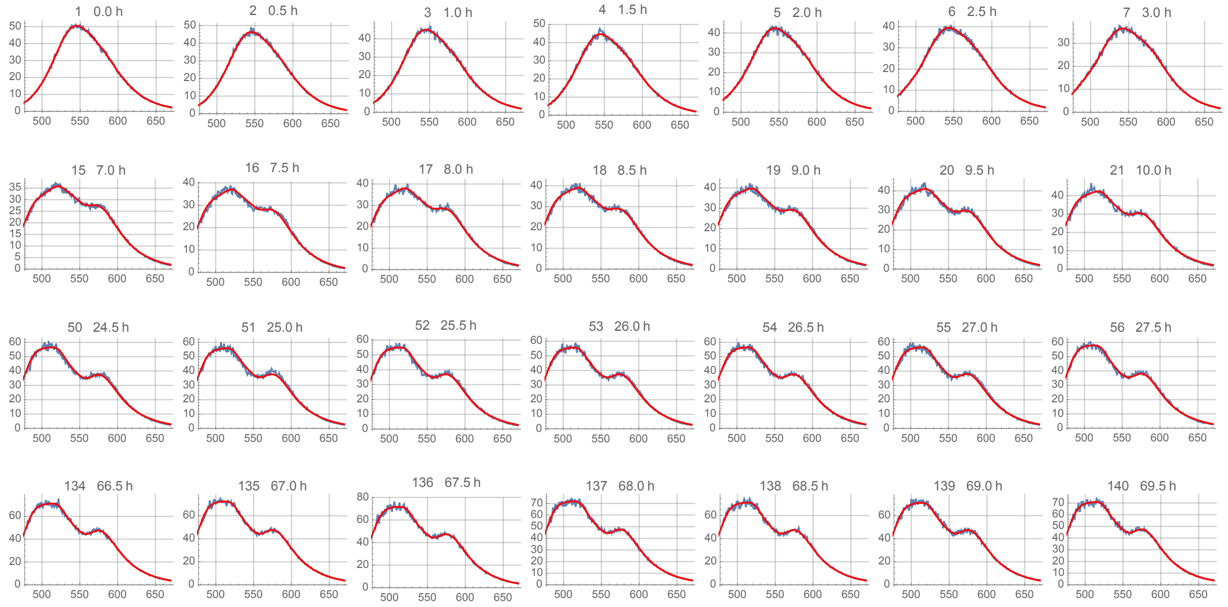

7. ***Kinetic model.*** The model reproduced from **Figure 8** of the main text is as follows:

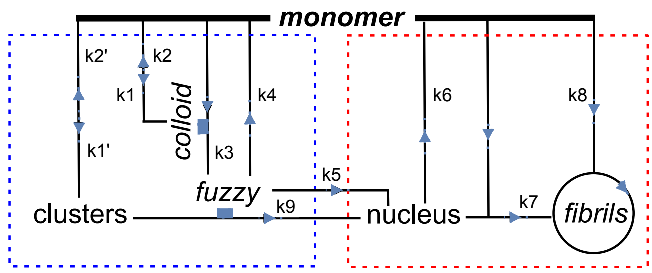

The differential equations corresponding to this scheme are:

```
m'[t] == -k1 n2 m[t]^n2 + k2 n2 m2[t] - k1a n1 m[t]^n1 + k2a n1 m1[t] - k3 p m[t]^p m2[t] + k4 p m3[t] + k6 p m4[t] - m[t] (k7 m4[t] + k8 m5[t]),
m1'[t] == k1a m[t]^n1 - k2a m1[t] - k9 m3[t] m1[t],
m2'[t] == k1 m[t]^n2 - k2 m2[t],
m3'[t] == k3 m[t]^p m2[t] - k4 m3[t] - k5 m3[t],
m4'[t] == k5 m3[t] - k6 m4[t] - k7 m[t] m4[t] + (n1/p) k9 m3[t] m1[t],
m5'[t] == m[t] (k7 (1 + p) m4[t] + k8 m5[t]),
m[0] == mtot, m1[0] == 0, m2[0] == 0, m3[0] == 0, m4[0] == 0, m5[0] == 0
m0 = m + n1 m1 + n2 m2 + p m3 + p m4 + m5
```

where m = monomer, m1 = cluster, m2 = *colloid*, m3 = *fuzzy*, m4 = nucleus, and m5 = *fibrils*, and m0 = total monomer concentration. Cluster, *colloid, fuzzy* and nucleus are regarded as *n*th order condensations of monomer; in the equations, the respective order parameters are n1, n2, p, and p. Simulations are performed typically with n1 = 10, n2 = 2, and p = 2. In the equations k1a = k1' and k2a = k2', where k1' and k2' are identified in **Figure 8**.

Convenient estimations of k1 and k1a are obtained from the formulation for the relaxation time τ of an isolated concerted *n*th order association (Bernasconi, 1976), often invoked for describing the nucleation of fibrillation (Saric, Michaels et al., 2016).

$$nA \underset{k_{-1}}{\overset{k_1}{\rightleftharpoons}} B$$

$$\tau^{-1} = n^2 k_1 c_A^{n-1} + k_{-1}$$

A preliminary assessment of the dependence of important reaction features in the individual parameters of the equations is given in Table S1. Examples of the complex influence of the key rate constant k3 are featured on Page 8.

Table S1. Influence of each rate constant on the performance of the scheme, Preliminary.

constant	*yield*					*fibrillation*			*comments*
	oligo	colloid	fuzzy	nucleus	fibril	$t_{1/2}$	slope	yield	
k1'	↑	↓*	↓	↓		↑	↓		*stabilizes colloid
k2'	↓	↑		↑		↑			
k1		↑	↑	↑			↓	↓	
k2		↓*	↓	↓		±↑	±↑		* shorter time course
k3	↓		↑			↑	↓	↓	has to be finite
k4			↓	↓					
k5	↓		↓	↓		↓			
k6									slight effect
k7									has to be finite
k8	↓			↑		↓	↑		very sensitive
k9	↓			*		↓	↑		* altered kinetics

8. *References*

Bernasconi CF (1976) Relaxation Kinetics. Academic Press,

Fauerbach JA, Yushchenko DA, Shahmoradian SH, Chiu W, Jovin TM, Jares-Erijman EA (2012) Supramolecular non-amyloid intermediates in the early stages of alpha-synuclein aggregation. Biophys J 102: 1127-36

Saric A, Michaels TCT, Zaccone A, Knowles TPJ, Frenkel D (2016) Kinetics of spontaneous filament nucleation via oligomers: Insights from theory and simulation. J Chem Phys 145: 211926

Yushchenko DA, Fauerbach J, Thirunavukkuarasu S, Jares-Erijman E, Jovin TM (2010) Fluorescent ratiometric MFC probe sensitive to early stages of alpha-synuclein aggregation. J Amer Chem Soc 132: 7860-7861

9. *Kinetics simulations* (parameter characterization). Shown on the next page.

5. Effect of ↑k3: decreases oligo and increases fuzzy, increases t1/2, decreases slope and yield

Model X3

n: 10 no: 2 p: 2 mtot: 150

k1: $1.\times 10^{-21}$ k1o: 0.0004 k2: 0.1 k2o: 0.1 k3: 0.00005 k4: 0.02 k5: 0.003 k6: 0.0001 k7: 0.1 k8: 0.003 k9: 0

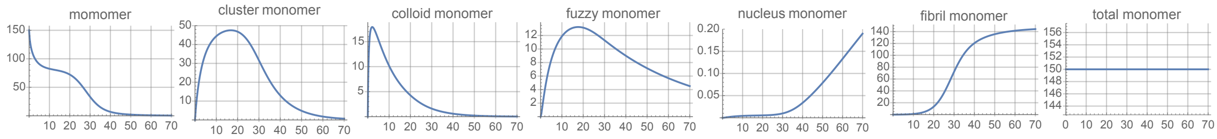

toh = 29.9911 foh = 72.0912 fmax = 144.182

thmtot = 30.3946

tslopemax = 28.5767 slopemax = 7.46329

Model X3

n: 10 no: 2 p: 2 mtot: 150

k1: $1.\times 10^{-21}$ k1o: 0.0004 k2: 0.1 k2o: 0.1 k3: 0.0005 k4: 0.02 k5: 0.003 k6: 0.0001 k7: 0.1 k8: 0.003 k9: 0

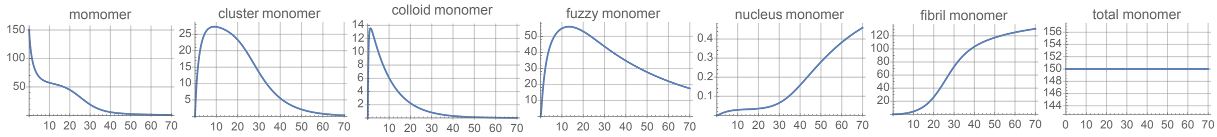

toh = 28.3926 foh = 65.3858 fmax = 130.772

thmtot = 30.5267

tslopemax = 25.8394 slopemax = 4.97544

Model X3

n: 10 no: 2 p: 2 mtot: 150

k1: $1.\times 10^{-21}$ k1o: 0.0004 k2: 0.1 k2o: 0.1 k3: 0.005 k4: 0.02 k5: 0.003 k6: 0.0001 k7: 0.1 k8: 0.003 k9: 0

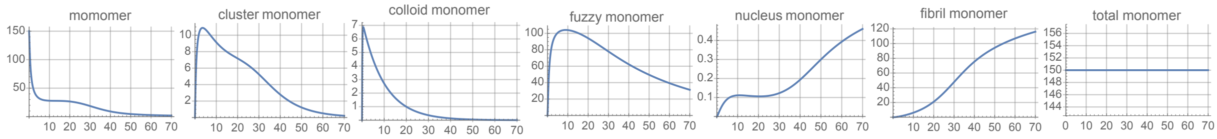

toh = 33.4614 foh = 58.0639 fmax = 116.128

thmtot = 39.8256

tslopemax = 30.3224 slopemax = 3.01679

Model X3

n: 10 no: 2 p: 2 mtot: 150

k1: $1.\times 10^{-21}$ k1o: 0.0004 k2: 0.1 k2o: 0.1 k3: 0.05 k4: 0.02 k5: 0.003 k6: 0.0001 k7: 0.1 k8: 0.003 k9: 0

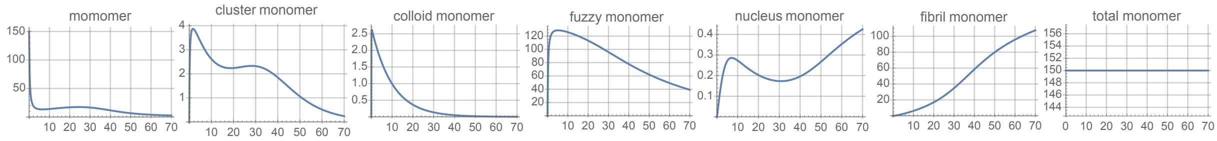

toh = 38.0636 foh = 53.7401 fmax = 107.48

thmtot = 47.4765

tslopemax = 37.1436 slopemax = 2.42504
